# Zebrafish Klf4 maintains the ionocyte progenitor population by regulating epidermal stem cell proliferation and lateral inhibition

**DOI:** 10.1371/journal.pgen.1008058

**Published:** 2019-04-01

**Authors:** Yi-Chung Chen, Bo-Kai Liao, Yu-Fen Lu, Yu-Hsiu Liu, Fang-Chi Hsieh, Pung-Pung Hwang, Sheng-Ping L. Hwang

**Affiliations:** 1 Institute of Cellular and Organismic Biology (ICOB), Academia Sinica, Taipei, Taiwan, Republic of China; 2 Department of Aquaculture, National Taiwan Ocean University, Keelung, Taiwan, Republic of China; 3 Department of Life Science, National Taiwan University, Taipei, Taiwan, Republic of China; 4 Graduate Institute of Life Sciences, National Defense Medical Center, Taiwan, Republic of China; Fred Hutchinson Cancer Research Center, UNITED STATES

## Abstract

In the skin and gill epidermis of fish, ionocytes develop alongside keratinocytes and maintain body fluid ionic homeostasis that is essential for adaptation to environmental fluctuations. It is known that ionocyte progenitors in zebrafish embryos are specified from p63^+^ epidermal stem cells through a patterning process involving DeltaC (Dlc)-Notch-mediated lateral inhibition, which selects scattered *dlc*^+^ cells into the ionocyte progenitor fate. However, mechanisms by which the ionocyte progenitor population is modulated remain unclear. Krüppel-like factor 4 (Klf4) transcription factor was previously implicated in the terminal differentiation of mammalian skin epidermis and is known for its bifunctional regulation of cell proliferation in a tissue context-dependent manner. Here, we report novel roles for zebrafish Klf4 in the ventral ectoderm during embryonic skin development. We found that Klf4 was expressed in p63^+^ epidermal stem cells of the ventral ectoderm from 90% epiboly onward. Knockdown or knockout of *klf4* expression reduced the proliferation rate of p63^+^ stem cells, resulting in decreased numbers of p63^+^ stem cells, *dlc*^*-*^p63^+^ keratinocyte progenitors and *dlc*^+^ p63^+^ ionocyte progenitor cells. These reductions subsequently led to diminished keratinocyte and ionocyte densities and resulted from upregulation of the well-known cell cycle regulators, *p53* and *cdkn1a*/*p21*. Moreover, mutation analyses of the KLF motif in the *dlc* promoter, combined with *VP16-klf4* or *engrailed-klf4* mRNA overexpression analyses, showed that Klf4 can bind the *dlc* promoter and modulate lateral inhibition by directly repressing *dlc* expression. This idea was further supported by observing the lateral inhibition outcomes in *klf4*-overexpressing or knockdown embryos. Overall, our experiments delineate novel roles for zebrafish Klf4 in regulating the ionocyte progenitor population throughout early stem cell stage to initiation of terminal differentiation, which is dependent on Dlc-Notch-mediated lateral inhibition.

## Author summary

The skin epidermis of terrestrial vertebrates is composed of a stratified epithelium, and requires krüppel-like factor 4 (KLF4) to establish a functional permeability barrier that protects animals from dehydration. In contrast, fish must tolerate variations in salinity and pH of the aquatic environment. As such, the fish skin epidermis is composed of keratinocytes and ionocytes, which transport ions and acid-base equivalents to maintain ionic and acid-base homeostasis of body fluids. In the present study, we used embryonic zebrafish as a model to investigate how Klf4 modulates the cell densities of matured ionocytes from early stem cell stages through the initiation of terminal differentiation by Dlc-Notch-mediated lateral inhibition. We showed that Klf4 promotes cell proliferation in epidermal stem cells, where it represses *p53* expression and prevents *cdkn1a/p21* induction. Additionally, Klf4 regulates the ionocyte progenitor population by directly repressing *dlc* expression and modulating lateral inhibition. Our findings describe novel roles for zebrafish Klf4 in ionocyte development and provide insights into the mechanism by which Klf4 regulates proliferation-differentiation balance in epidermal stem cells.

## Introduction

Unlike terrestrial vertebrates, teleosts encounter and adapt to ionic, osmotic and acid-base fluctuations in aquatic environments. To maintain body fluid ionic homeostasis, specialized ionocytes (previously called mitochondria-rich cells) develop predominantly in the skin of embryos and gills of adult fish. These cells regulate osmotic homeostasis through transepithelial ion-transport [1, 2]. Five types of ionocytes have been identified in the skin of zebrafish embryos, including H^+^-ATPase-rich (HR) cells, Na^+^, K^+^-ATPase-rich (NaR) cells, Na^+^-Cl^-^ cotransporter-expressing (NCC) cells, SLC26-expressing cells, and K^+^-secreting (KS) cells [3]. These ionocytes perform transepithelial H^+^ secretion/Na^+^ uptake/NH4^+^ excretion, Ca^2+^ uptake, Na^+^/Cl^-^ uptake, Cl^-^ uptake/HCO_3_^-^ secretion, and K^+^ secretion, respectively, by utilizing various transporters located at the apical or basolateral cell surface [3–6].

In zebrafish embryos, ionocytes and keratinocytes are derived from common precursors in the ventral non-neural ectoderm and express a dominant-negative form of p63 (ΔNp63) at the late gastrula stage [7]. ΔNp63 was shown to be a direct target of BMP signaling, which is essential to promote ectodermal cell differentiation into epidermal cells [8]. The functional consequences of Δ*Np63* were revealed in a study that showed reduced expression of *gata2* non-neural marker and enhanced expression of neuroectoderm markers (*six3*.*1* and *pax2*.*1*) during gastrulation, suggesting an early role for ΔNp63 as a repressor of neural specification in the ventral ectoderm. In addition, Δ*Np63* morphants were reported to have lost fin fold and pectoral fins, which was attributed to defective p53 inhibition. Thus, ΔNp63 also plays a late role, after 20 hours post fertilization (hpf), in the maintenance of epidermal proliferation [8, 9]. Although ionocyte progenitors transiently express Δ*Np63* from the bud to the 14 somite stage, knockdown of Δ*Np63* does not abolish proliferation or differentiation of ionocytes [7, 10], indicating that p63 is unlikely to be a master regulator of proliferation control in ionocyte progenitors.

Delta-Notch-mediated lateral inhibition determines whether cells from the p63^+^ ventral ectoderm will become ionocytes or keratinocytes [7, 10]. Epidermal cells expressing high levels of Dlc ligand become ionocyte progenitors, and Dlc binding to Notch1a/3 receptors on neighboring epidermal cells prevents them from adopting the same cell fate. Without *dlc* expression, the neighboring cells develop into keratinocytes [10]. Lateral inhibition is widely known to be regulated by signaling strength, and a recent study on *Drosophila* sensory organ precursor (SOP) cells showed that cellular proliferation also plays a crucial role in determining lateral inhibition-controlled tissue patterning [11]. In addition to Notch signaling strength and proliferation rates, there are several other potential mechanisms by which lateral inhibition may be modulated. These mechanisms include effects on the extent of the lateral inhibition domain, or control of *delta* expression levels after cell division. These multiple control processes may be influenced by a master regulator that differentially modulates epidermal stem cells and ionocyte progenitors during lateral inhibition, however, such a master regulator has not yet been identified.

After the progenitor cell fate is determined by lateral inhibition, expression of two winged helix/forkhead transcription factors (*foxi3a* and *foxi3b*) can be observed in ionocyte progenitors during late gastrulation. Knockdown of *foxi3a* abolishes the development of ionocytes, including HR and NaR cells, indicating a requirement for Foxi3a in the specification and differentiation of ionocytes. Furthermore, a positive feedback regulatory loop between Foxi3a and Foxi3b is thought to control specification into different ionocyte subtypes [10]. This loop creates individual expression profiles for the two proteins that differentially regulate downstream determination factors, such as *glial cell missing 2* (*gcm2*), to specify ionocyte progenitors into HR cells or NaR cells [12, 13]. Thus, a self-organized and evenly distributed pattern of ionocytes emerges in the epidermal tissue, and factors that influence ionocyte progenitor proliferation would be expected to affect all types of matured ionocytes.

Mammalian krüppel like factor 4 (KLF4) is a zinc-finger transcription factor [14] that is composed of an N-terminal activation domain, a central repression domain, and three zinc-finger DNA binding motifs at the C-terminus. The protein is expressed and functions in a variety of tissues, including intestinal epithelium and skin [15, 16]. Interestingly, *Klf4*^*-/-*^ mice die shortly after birth because of defects in the skin barrier function [17]. An epidermal permeability barrier consists of several layers, including the outer stratum corneum, which is composed of a cornified envelope and lipid bilayers; it is the development of the cornified envelope that is selectively affected in *Klf4*^*-/-*^ mice [17, 18]. Mechanistically, KLF4 positively regulates expression of *Sprr2a*, which encodes a proline-rich protein in the cornified envelope, and nine different *Keratin* genes, which form keratin filaments. Thus, KLF4 is known to be essential for terminal differentiation of skin epidermis [17, 19].

KLF4 also acts as an oncogene or tumor suppressor in a context-dependent manner [20, 21]. The tumor suppressor activity is related to induction of cell cycle arrest via transcriptional upregulation of the *CDKN1A* gene, which encodes p21^Cip1^. Correspondingly, gastric epithelia in mice with *Klf4* deficiency exhibit low levels of p21^Cip1^ expression, resulting in increased proliferation [22, 23]. Conversely, KLF4 may act as an oncogene by binding to the promoter of *p53* and suppressing transcription of the gene [24]. In cells expressing RAS^V12^, retroviral delivery of KLF4 promoted proliferation through repression of p53 and inactivation of p21^Cip1^ via Cyclin D1 inhibition [24]. In addition, KLF4 was shown to regulate embryonic stem cell self-renewal by directly enhancing *Nanog* expression to prevent differentiation [25]. Thus, the cellular functions of KLF4 are multifaceted, and it is still not clear how KLF4 regulates the balance of epidermal proliferation-differentiation.

Here, we report novel roles for zebrafish Klf4 in the maintenance of the ionocyte progenitor population by regulating epidermal stem cell proliferation and modulating *dlc*-mediated lateral inhibition. In order to examine ionocyte development in zebrafish embryos, we generated *dlc* transgenic lines that recapitulate endogenous *dlc* expression. Strikingly, *dlc*^+^ ionocyte progenitor cells were absent in mutant lines with a defective KLF binding motif on the *dlc* promoter, which was identified by chromatin immunoprecipitation (ChIP). Furthermore, we found that Klf4 is expressed universally in p63^+^ epidermal stem cells located in the ventral ectoderm from 90% epiboly. Knockdown or knockout of *klf4* expression reduced the proliferation rate of p63^+^ stem cells, resulting in decreased numbers of p63^+^ stem cells, *dlc*^*+*^ p63^+^ionocyte and *dlc*^-^p63^+^ keratinocyte progenitors. These decreased numbers led to subsequent decreases in the densities of HR and NaR ionocytes, as well as *col1a1a*^*+*^ keratinocytes. We found that Klf4 modulated the ionocyte progenitor population through multiple mechanisms, including p53-mediated effects on proliferation of p63^+^ epidermal stem cells and ionocytes, regulating the range of lateral inhibition domain, repressing *dlc* expression and affecting *dlc*^+^ progenitor clustering.

## Results

### Zebrafish Klf4 regulates the proliferation of p63^+^ stem cells and *dlc*^+^ ionocyte progenitor cell number

**Fig 1:**
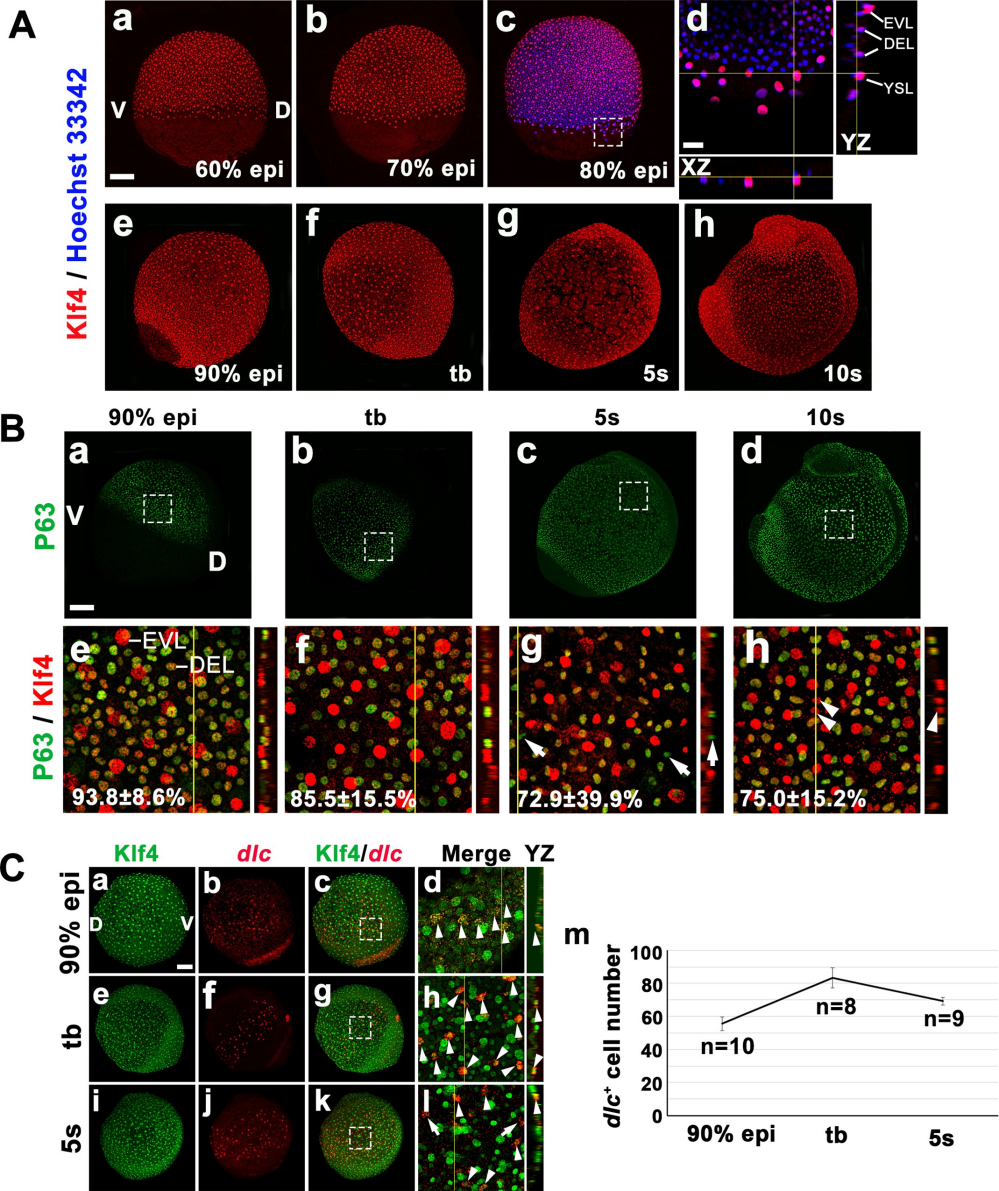
Klf4 protein expression pattern and colocalization of Klf4/p63 and Klf4/*dlc* during late gastrulation and early somite stages. (**A**) Klf4 is expressed in the epiblast of the deep cell layer (DEL), yolk syncytial layer (YSL), and enveloping layer cells (EVL) of 60%, 70%, 80% and 90% epiboly embryos. Insets show respective XZ or YZ projections of confocal images from an 80% epiboly embryo along the axes shown in the main panel (d). Klf4 is expressed in the ectoderm and EVL of bud (tb), 5s and 10s embryos. (**B**) Images of p63 expression during different embryonic developmental stages are shown (a-d). Colocalization of Klf4 and p63 was detected in ventral ectoderm of embryos at various stages, including 90% epiboly, bud, 5s and 10s (e-h). Enlargements of Klf4 and p63 merged images (e-h) are shown from the areas indicated in lower magnification images (a-d). Percentage of colocalization of Klf4 and p63 in respective stage is indicated within each panel. Some non-colocalized cells showing only expression of Klf4 (arrowhead) or p63 (arrow) were observed in the ventral ectoderm of embryos at 5s and 10s. Insets show YZ projections of confocal images. (**C**) Images of wild-type embryos stained with anti-Klf4 antibody and *dlc* antisense RNA at 90% epiboly, bud and 5s stages are shown. Enlargements of merged images (d, h, l) show the areas indicated in corresponding lower magnification pictures (c, g, k). Insets show YZ projections from confocal images. Arrowheads indicate examples of ionocyte progenitors with colocalized Klf4 and *dlc* in the epidermal ionocyte domain. Arrows indicate ionocyte progenitors expressing only *dlc*. *dlc*^+^ ionocyte progenitor number at indicated embryonic stages is shown (m). Scale bar, 50 μm. Error bars indicate standard error.

Previously, we demonstrated that zebrafish Klf4 plays an evolutionarily conserved role in regulating the differentiation of intestinal goblet cells, much like its counterpart, KLF4, in mouse [26]. Because mouse KLF4 also plays an essential role in the terminal differentiation of skin epidermis, we investigated whether zebrafish *klf4* is expressed in the epidermis and affects the development of ionocytes in embryos. Immunofluorescence was conducted using an anti-zebrafish Klf4 antibody, and Klf4 was found to be universally expressed in the epiblast of the deep cell layer (DEL), yolk syncytial layer (YSL), and enveloping layer cells (EVL) during gastrulation. Moreover, Klf4 staining was observed in both ventral and dorsal ectoderm, and EVL cells during early somite stages (Fig 1A).

Epidermal stem cells are marked by p63 [27], and are known to give rise to ionocyte progenitors [10]. We examined the distribution pattern of p63 and Klf4 by double immunofluorescence in stages ranging from late gastrulation to early somite. p63 expression was first observed in the ventral ectoderm of 90% epiboly and bud stage embryos, where it was highly colocalized with Klf4 (93.8 ± 8.6% in 90% epiboly and 85.5 ± 15.5% in bud, mean ± SEM) (Fig 1B). Expanded p63 expression in the dorsal ectoderm was found in 5s and 10s embryos, and some Klf4 positive cells did not colocalize with p63 epidermal stem cells in the ventral ectoderm region at the stages screened (Fig 1B). Those cells that only express Klf4 are not expected to be ionocyte precursors, because p63 was consistently colocalized with ionocyte precursors until at least the 14s stage [10]. In addition, colocalization of Klf4 protein and *dlc* mRNA was observed beginning at 90% epiboly until the bud stage, and all *dlc*^+^ cells also stained positive for Klf4 (Fig 1Ca-h). At the 5s stage, some *dlc*^+^ cells in the epidermal ionocyte domain (yolk ball) were not labeled by Klf4 staining (Fig 1Ci-l). *dlc*^+^ ionocyte progenitor number reached a maximum at bud stage and was decreased at the 5s stage (Fig 1Cm). This result suggests that the selection of *dlc*^+^ ionocyte progenitors via lateral inhibition occurs at the bud stage and that *dlc* degradation is initiated between the bud and 5s stage.

**Fig 2:**
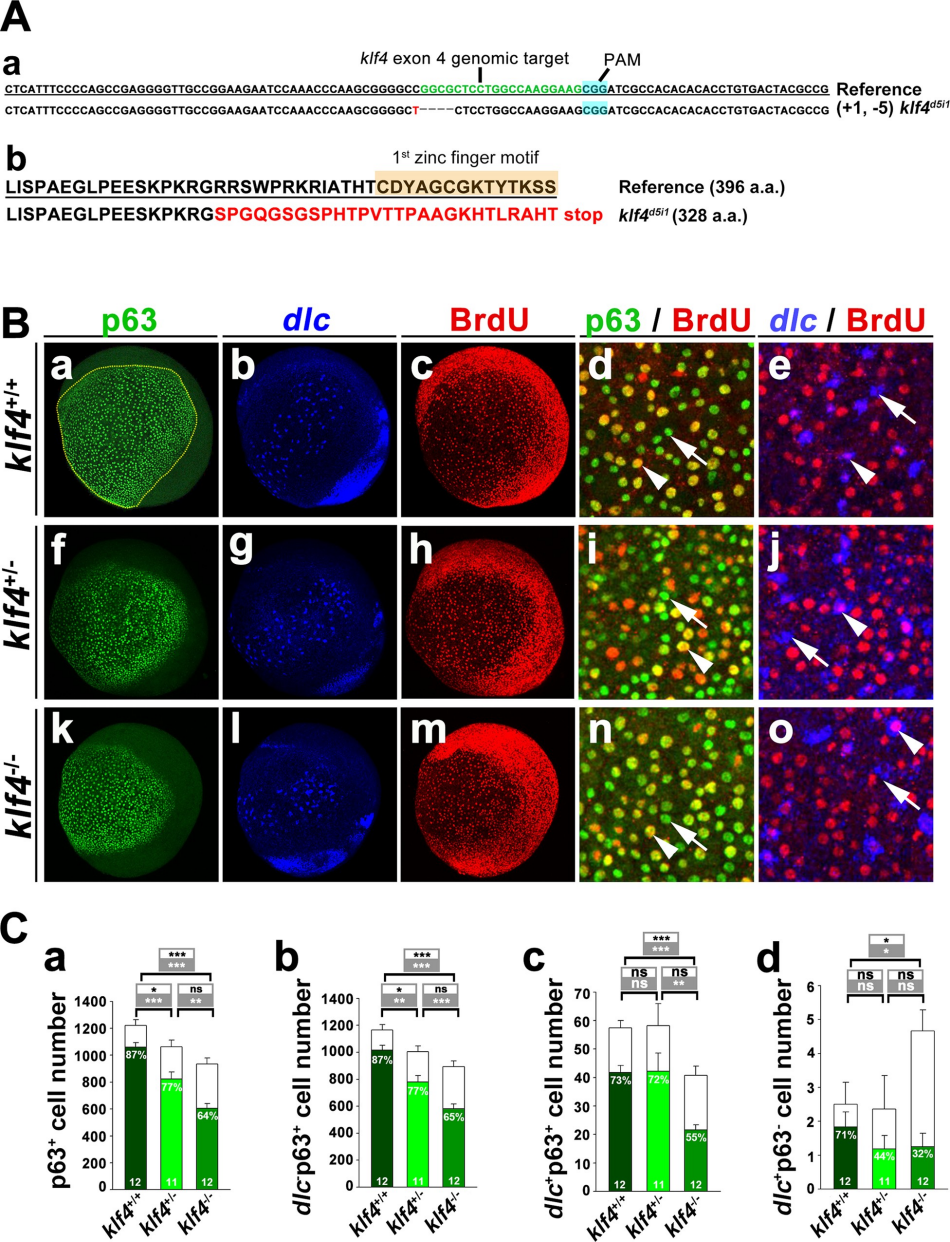
Knockout of *klf4* reduced proliferation of epidermal stem cells and *dlc*^+^ ionocyte progenitor cell number. **(A)** (a) Partial nucleotide sequence of wild-type (reference) *klf4*, showing sgRNA (green lettering) targeted to exon 4 is shown. The *klf4*^*d5i1*^ mutant had a 1 bp insertion (red lettering) and 5 bp deletion (dashed line). Protospacer adjacent motif (PAM) sequence is shown in blue. (b) Predicted amino acid sequence of *klf4* with the first zinc finger motif in wild type is compared to the misframed zinc finger domain and stop codon (red lettering) in *klf4*^*d5i1*^ mutants. **(B)** Images of BrdU-labeled *klf4*^+/+^, *klf4*^+/-^ or *klf4*^-/-^ embryos, followed by staining with *dlc* antisense RNA, and stained with anti-p63 and anti-BrdU antibodies at bud stage are shown. Both p63^+^ and p63^+^ BrdU^+^ cell number were enumerated in the circled area of *klf4*^+/+^, *klf4*^+/-^ or *klf4*^-/-^ embryos. Enlarged images of *klf4*^+/+^, *klf4*^+/-^ or *klf4*^-/-^embryos stained with p63 and BrdU or *dlc* RNA probe and BrdU are shown. Examples of p63 and BrdU or *dlc* and BrdU colocalized (arrowhead) or non colocalzed (arrow) cells are shown. (**C**) Quantitative results from (B). Total p63^+^ or *dlc*^-^ p63^+^ cell numbers (open bars) with BrdU^+^ cell numbers (filled bar) of *klf4*^+/+^, *klf4*^+/-^ or *klf4*^-/-^ embryos at bud stage are shown in (a) and (b). *dlc*^*+*^p63^+^ or *dlc*^*+*^p63^-^ cell numbers (open bars) with BrdU^+^ cell numbers (filled bars) of *klf4*^+/+^, *klf4*^+/-^ or *klf4*^-/-^ embryos at bud stage are shown in (c) and (d). Statistical significance is indicated for comparisons of total cell numbers (open box) or BrdU^+^ cell numbers (filled box). Individual percentages of p63^+^BrdU^+^, *dlc*^-^ p63^+^ BrdU^+^, *dlc*^+^ p63^+^ BrdU^+^ or *dlc*^+^ p63^-^ BrdU^+^ cells of *klf4*^+/+^, *klf4*^+/-^ or *klf4*^-/-^ embryos at bud stage are indicated within the bar. Embryos are shown in lateral view. Statistical significance was determined by Student’s *t*-test. NS, not significant; *p < 0.05; **p < 0.01; ***p < 0.001. Error bars indicate standard error.

Since mammalian KLF4 is known to regulate embryonic stem cell self-renewal, we wondered whether zebrafish Klf4 might modulate p63^+^ epidermal stem cell proliferation and *dlc*^+^ ionocyte progenitor number. Thus, we knocked out *klf4* by CRISPR-Cas9 genomic editing [28]. Four sgRNAs were designed to target exon 4 at a position 5'-upstream of the first zinc finger motif, but only one of the sgRNAs efficiently generated a new mutant strain, which was named *klf4*^*d5i1*^ and contained an indel consisting of a 5-bp deletion and a 1-bp insertion. This mutant produces a truncated Klf4 protein that consists of 328 amino acids, 27 of which are misframed, and no functional zinc finger motif (Fig 2A). We labeled 80% epiboly *klf4*^*d5i1*^ F3 embryos with BrdU and fixed the embryos at the bud stage. Immunofluorescence staining was performed, using anti-BrdU and anti-p63 antibodies, in combination with fluorescence *in situ* hybridization, using a *dlc* antisense RNA probe (Fig 2B). Each *klf4*^*d5i1*^ F3 embryo was genotyped after imaging. The p63^+^ epidermal stem cell number was reduced by 13.1% in *klf4*^*d5i1*^ heterozygous mutants (*klf4*^*+/+*^ 1222.5 ± 41.7 cells vs. *klf4*^*+/-*^ 1062.3 ± 49.2 cells; *t*-test, *p* = 9.5 × 10^−4^) and 23.5% in *klf4*^*d5i1*^ homozygous mutant embryos (*klf4*^*+/+*^ 1222.5 ± 41.7 cells vs. *klf4*^*-/-*^ 935 ± 43.6 cells; *t*-test, *p* = 5.8 × 10^−9^) compared to sibling wild-type controls at the bud stage (Fig 2Bf,k and 2Ca). These reductions were attributable to a decreased proportion of p63^+^ BrdU^+^ epidermal stem cells in *klf4*^*d5i1*^ heterozygous and homozygous mutant embryos at the bud stage (Fig 2Bi,n and 2Ca), suggesting that Klf4 is required to maintain the proliferation rate of p63^+^ epidermal stem cells.

Since p63^+^ epidermal stem cells can produce both keratinocytes and ionocytes, we then analyzed proliferation and numbers of *dlc*^*-*^p63^+^ cells (keratinocyte precursors) and *dlc*^*+*^p63^+^ cells (ionocyte precursors) in *klf4*^*d5i1*^ heterozygous, homozygous mutant and sibling wild-type embryos by analyzing merged images of embryos stained for p63 and *dlc*. *dlc*^-^ p63^+^ keratinocyte progenitor cell number was decreased by 13.8% in *klf4*^*d5i1*^ heterozygous mutant embryos (*klf4*^*+/+*^ 1165.1 ± 41.2 cells vs. *klf4*^*+/-*^ 1004 ± 43.5 cells; *t*-test, *p* = 6.853 × 10^−3^) and 23.2% in *klf4*^*d5i1*^ homozygous mutant embryos (*klf4*^*+/+*^ 1165.1 ± 41.2 cells vs. *klf4*^*-/-*^ 894.25 ± 41.9 cells; *t*-test, *p* = 1.36 × 10^−4^) compared to sibling wild-type controls (Fig 2Cb). The percentage of *dlc*^-^ p63^+^ BrdU^+^ keratinocyte progenitor cells was decreased by 11.5% in *klf4*^*d5i1*^ heterozygous embryos (*klf4*^*+/+*^ 87.3 ± 0.81% vs. *klf4*^*+/-*^ 77.3 ± 2.26%; *t*-test, *p* = 1.06 × 10^−3^) and 25.3% in *klf4*^*d5i1*^ homozygous mutants (*klf4*^*+/+*^ 87.3 ± 0.81% vs. *klf4*^*-/-*^ 64.7 ± 1.5%; *t*-test, *p* = 2.18 × 10^−10^) compared to wild types (Fig 2Cb).

*dlc*^+^ p63^+^ ionocyte progenitor cell number was also decreased in *klf4*^*d5i1*^ homozygous mutant embryos compared to wild-type siblings (*klf4*^*+/+*^ 57.41 ± 2.6 cells vs. *klf4*^*-/-*^ 40.75 ± 3.28 cells; *t*-test, *p* = 6.31 × 10^−4^), however, no reduction of *dlc*^+^ p63^+^ ionocyte progenitor cell number was detected in *klf4*^*d5i1*^ heterozygous mutants at bud stage (Fig 2Cc). Consequently, a reduced (24.7%) proportion of *dlc*^+^ p63^+^BrdU ^+^ cells was identified in *klf4*^*d5i1*^ homozygous (*klf4*^*+/+*^ 72.5±2.66% vs *klf4*^*-/-*^54.7±3.79%; *t*-test, *p* = 7.93 × 10^−4^) mutant, but not in *klf4*^*d5i1*^ heterozygous mutant embryos at bud stage (Fig 2Cc). Surprisingly, we also identified a small number (< 5) of *dlc*^+^ p63^-^ cells in all examined genotypes (Fig 2Cd), which we suspect resulted from erroneous labeling (Fig 2Cd).

We also knocked down *klf4* by antisense morpholino oligomers (*klf4* MO1 and *klf4* MO2), which have been previously validated for specificity and efficacy [26]. Significant decreases in the total number of p63^+^ epidermal stem cells, which may be attributed to reduced proliferation rate, were identified in *klf4* morphants compared to control embryos at the bud stage (S1Ca Fig). Similar declines in *dlc*^-^ p63^+^ keratinocyte progenitor cell number and proliferation rate were detected in *klf4* morphants compared to control embryos (S1Cb Fig). A substantially reduced number of *dlc*^+^ p63^+^ ionocyte progenitor cells was found in *klf4* morphants, however the proliferation rate was not altered (S1Cc Fig). We also identified a small number (< 5) of *dlc*^+^ p63^-^ cells in control and *klf4* morphants, which were probably the result of erroneous labeling (S1Cd Fig). Together, these results indicate that Klf4 regulates the proliferation rate of p63^+^ epidermal stem cells and *dlc*^-^ p63^+^ keratinocyte progenitor cells, as well as *dlc*^+^ p63^+^ ionocyte progenitor cell number.

### Zebrafish Klf4 regulates the differentiation of epidermal ionocytes

**Fig 3:**
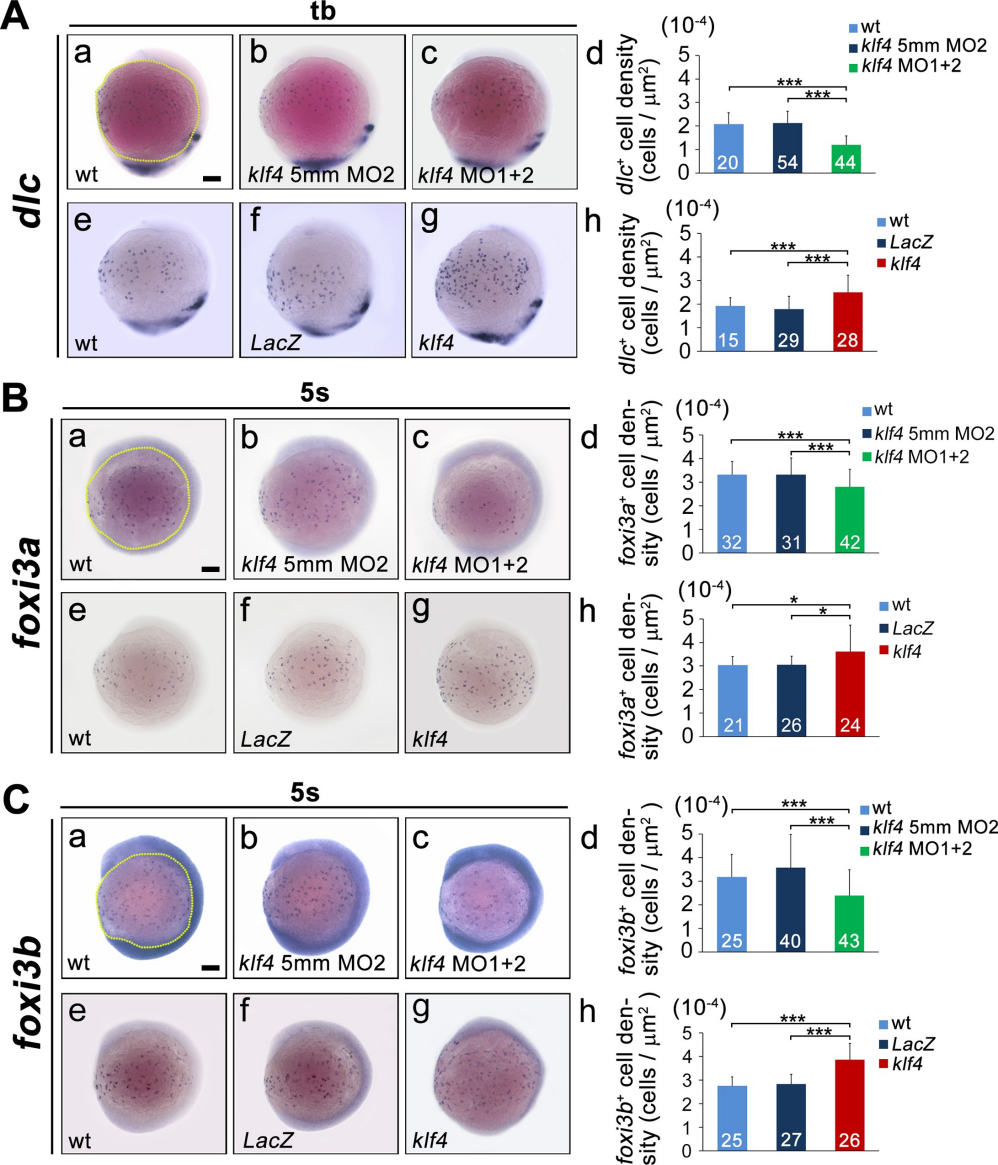
Altered *klf4* expression at bud or 5s stages affects cell density of ionocyte progenitors expressing *dlc*, *foxi3a* or *foxi3b*. (**A**) The cell density of *dlc*^*+*^ ionocyte progenitors was reduced in embryos injected with both *klf4* MO1 and MO2 (c) as compared to uninjected wild type (a) or control embryos injected with *klf4* 5mmMO2 (b) at bud stage. Cell densities of *dlc*^*+*^ ionocyte progenitors were quantified from the indicated area in wild-type embryos and embryos injected with *klf4* 5mmMO2 or both *klf4* MO1 and MO2 (d). The density of *dlc*^*+*^ ionocyte progenitors was increased in *klf4*-overexpressing embryos (g) compared to *LacZ*-overexpressing (f) or uninjected wild-type (e) embryos. Cell density of *dlc*^*+*^ ionocyte progenitors was quantified in wild-type embryos and *LacZ* or *klf4* mRNA-injected embryos (h). (**B**) The density of *foxi3a*^*+*^ ionocyte progenitors was reduced in embryos injected with both *klf4* MO1 and MO2 (c), as compared to uninjected wild-type (a) or control embryos injected with *klf4* 5mmMO2 (b) at 5s stage. Cell densities of *foxi3a*^*+*^ ionocyte progenitors were quantified from the indicated area in wild-type embryos and embryos injected with *klf4* 5mmMO2 or both *klf4* MO1 and MO2 (d). The density of *foxi3a*^*+*^ ionocyte progenitors was increased in *klf4*-overexpressing embryos (g), as compared to *LacZ*-overexpressing (f) or uninjected wild-type (e) embryos. Cell densities of *foxi3a*^*+*^ ionocyte progenitors were quantified in wild-type embryos and *LacZ*- or *klf4*-injected embryos (h). (**C**) The density of *foxi3b*^*+*^ ionocyte progenitors was reduced in embryos injected with both *klf4* MO1 and MO2 (c) as compared to uninjected wild-type (a) or control embryos injected with *klf4* 5mmMO2 (b) at 5s stage. Cell density of *foxi3b*^*+*^ ionocyte progenitors was quantified from the indicated area in wild-type embryos and embryos injected with *klf4* 5mmMO2 or both *klf4* MO1 and MO2 (d). The density of *foxi3b*^*+*^ ionocyte progenitors was increased in *klf4*-overexpressing embryos (g) compared to *LacZ*-overexpressing (f) or uninjected wild-type (e) embryos. Cell density of *foxi3b*^*+*^ ionocyte progenitors was quantified in wild-type embryos and *LacZ* or *klf4* mRNA injected embryos (h). Embryos are shown in lateral view. Statistical significance was determined by Student’s *t*-test. *p < 0.05; ***p < 0.001. Scale bar, 100 μm. Error bars indicate standard error.

In order to confirm the effect of *klf4* deficiency on the *dlc*^+^ ionocyte progenitor cell number, we knocked down *klf4* by antisense morpholino oligomers (*klf4* MO1 and *klf4* MO2). We detected a significant decrease in the cell density of *dlc*^*+*^ ionocyte progenitors (control 2.13 ± 0.067 cells μm^-2^ × 10^−4^; morphants 1.20 ± 0.058 cells μm^-2^ × 10^−4^; *t*-test, *p* = 1.42 × 10^−17^) in embryos co-injected with *klf4* MOs as compared with control embryos. We further detected a substantial increase in *dlc*^*+*^ progenitor cell density (*LacZ*-overexpression 1.78 ± 0.10 cells μm^-2^ × 10^−4^; *klf4*-overexpression 2.50 ± 0.14 cells μm^-2^ × 10^−4^; *t*-test, *p* = 1.15 × 10^−4^) in *klf4*-overexpressing embryos at the bud stage (Fig 3A). Since the Foxi3a and Foxi3b winged helix/forkhead box transcription factors are master regulators of epidermal ionocyte specification in zebrafish embryos [10], we further investigated whether expression of these genes is affected by perturbing *klf4* levels. A significant reduction (control 3.31 ± 0.13 cells μm^-2^ × 10^−4^; morphants 2.80 ± 0.12 cells mm^-2^ × 10^−4^; *t*-test, *p* = 4.51 × 10^−3^ for *foxi3a*^+^ cells; control 3.12 ± 0.087 cells μm^-2^ × 10^−4^; morphants 2.60 ± 0.09; *t*-test, *p* = 9.72 × 10^−5^ for *foxi3b*^+^ cells) in the densities of ionocyte progenitors expressing either *foxi3a* or *foxi3b* was observed in embryos that were co-injected with *klf4* MOs as compared to control embryos at the 5s stage. Conversely, statistically significant increases in cell density of *foxi3a*^+^ or *foxi3b*^+^ ionocyte progenitors were observed (*LacZ*-overexpression 3.04 ± 0.071 cells μm^-2^ × 10^−4^; *klf4*-overexpression 3.61 ± 0.23 cells μm^-2^ × 10^−4^; *t*-test, *p* = 0.0279 for *foxi3a*^+^ cells; *LacZ*-overexpression 2.84 ± 0.07 cells μm^-2^ × 10^−4^; *klf4*-overexpression 3.87 ± 0.14 cells μm^-2^ × 10^−4^; *t*-test, *p* = 1 × 10^−7^ for *foxi3b*^+^ cells) in 5s embryos that were injected with *klf4* mRNA, as compared to embryos injected with *LacZ* mRNA (Fig 3B and 3C).

We also analyzed the densities of *dlc*^*+*^ and *foxi3a*^*+*^ ionocyte progenitor cells in *klf4*^*d5i1*^ homozygous mutant embryos. We observed a significant reduction in *dlc*^*+*^ ionocyte cell density (control 2.17 ± 0.010 cells μm^-2^ × 10^−4^; mutants 1.51 ± 0.033 cells μm^-2^ × 10^−4^; *t*-test, *p* = 0.01396) of *klf4*^*d5i1*^ homozygous mutant embryos compared to wild-type controls at bud stage (S2C Fig). Similarly, a substantial decrease of *foxi3a*^*+*^ ionocyte cell density (control 2.07 ± 0.11 cells μm^-2^ × 10^−4^; mutants 1.77 ± 0.055 cells μm^-2^ × 10^−4^; *t*-test, *p* = 0.03901) was detected in *klf4*^*d5i1*^ homozygous mutant embryos compared to wild-type controls at 5s stage (S2F Fig). Together these results confirm that defective *klf4* expression affects ionocyte progenitors, as evidenced by expression of ionocyte regulators, *dlc*, *foxi3a* and *foxi3b*.

**Fig 4:**
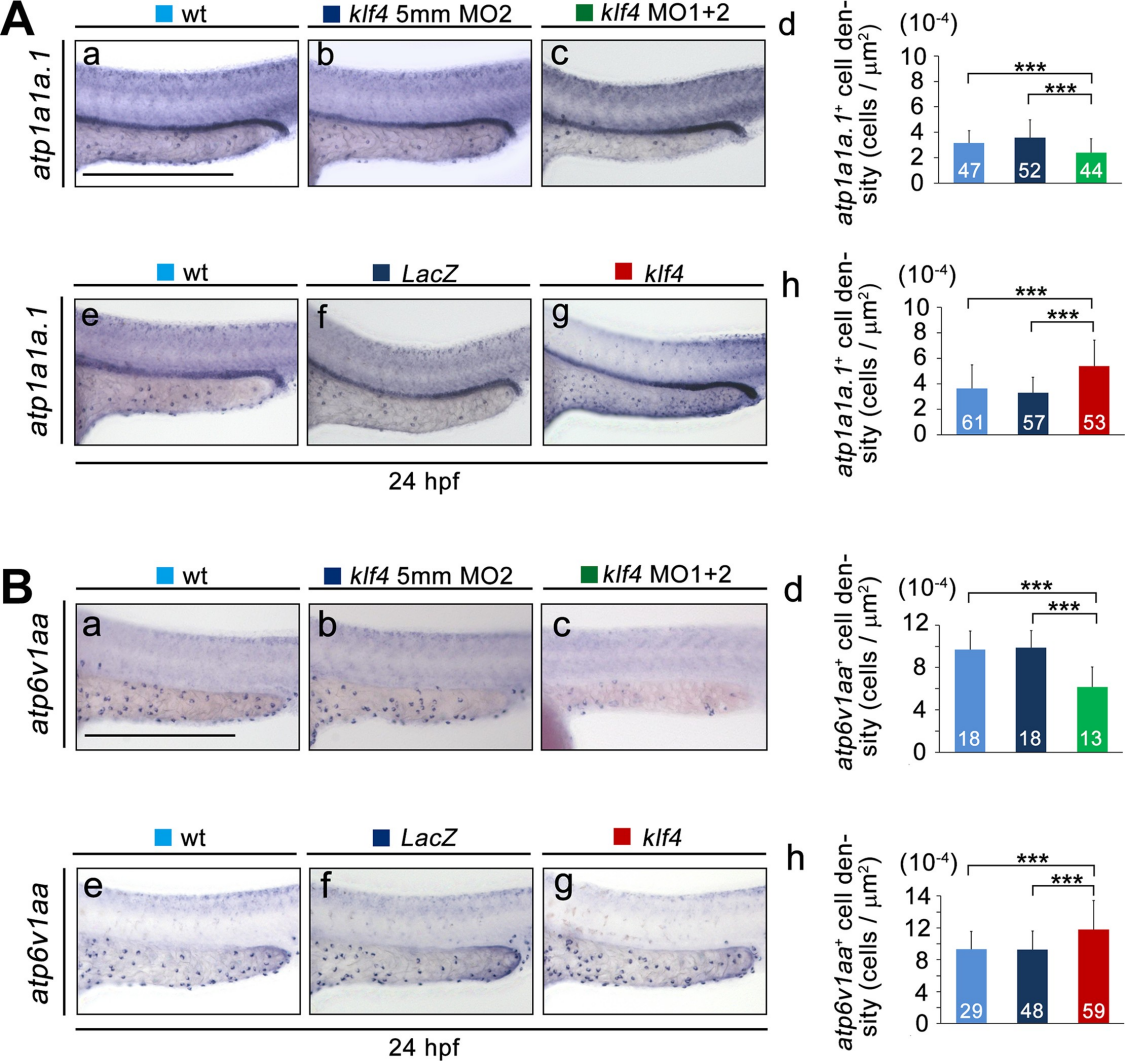
Perturbation of *klf4* expression affects the densities of NaR and HR cells at 24 hpf. (**A**) The density of *atp1a1a*.*1*^*+*^-Na^*+*^-K^*+*^-ATPase-rich (NaR) cells was reduced in yolk extensions of embryos injected with both *klf4* MO1 and MO2 (c) as compared to uninjected wild types (a) and control embryos injected with *klf4* 5mmMO2 (b). The density of *atp1a1a*.*1*^*+*^-NaR cells was increased in yolk extensions of embryos injected with *klf4* (g) mRNA, as compared to embryos injected with the same amount of *LacZ* (f) mRNA, or uninjected wild type (e) embryos. Quantification of results from (a-c) and (e-g) are shown in (d) and (h), respectively. (**B**) The density of *atp6v1aa* ^*+*^- H^*+*^-ATPase-rich (HR) cells was reduced in yolk extensions of embryos injected with both *klf4* MO1 and MO2 (c) as compared to uninjected wild types (a) and control embryos injected with *klf4* 5mmMO2 (b). The density of *atp6v1aa* ^*+*^-HR cells was increased in yolk extensions of embryos injected with *klf4* (g) mRNA, as compared to embryos injected with the same amount of *LacZ* (f) mRNA, or uninjected wild-type (e) embryos. Quantification of results from (a-c) and (e-g) are shown in (d) and (h), respectively. Statistical significance was determined by Student’s *t*-test. ***p < 0.001. Scale bar, 300 μm. Error bars indicate standard error.

Because NaR and HR cells have been studied most extensively among the five types of ionocytes, we next evaluated whether differentiation of NaR and HR cells was affected by perturbation of *klf4* expression. Expression of relevant marker genes (*atp1a1a*.*1* and *atp6v1aa* for NaR and HR cells, respectively) at 24 hpf was measured by *in situ* hybridization. A substantial decrease in *atp1a1a*.*1*^*+*^ NaR cell density (control 3.58 ± 0.19 cells μm^-2^ × 10^−4^; morphants 2.40 ± 0.16 cells μm^-2^ × 10^2^; *t*-test, *p* = 1.05 × 10^−5^) was found in 24 hpf embryos co-injected with *klf4* MOs, as compared with wild-type and control embryos injected with *klf4* 5mm MO2. Conversely, a significant increase in *atp1a1a*.*1*^*+*^ NaR cell density (3.31 ± 0.16 cells μm^-2^ × 10^−4^ for *LacZ*-overexpression; 5.40 ± 0.28 cells μm^-2^ × 10^−4^ for *klf4*-overexpression; *t*-test, *p* = 5.08 × 10^−9^) was observed in embryos injected with *klf4* mRNA at the same time-point (Fig 4A). A similar effect on *atp6v1aa*^*+*^ HR cell density was observed in *klf4* morphants (control 9.87 ± 0.37 cells μm^-2^ × 10^−4^; morphants 6.16 ± 0.53 cells μm^-2^ × 10^−4^; *t*-test, *p* = 1.99 × 10^−6^) and *klf4*-overexpressing embryos (9.28± 0.34 cells μm^-2^ × 10^−4^ for *LacZ*-overexpression; 11.82 ± 0.47 cells μm^-2^ × 10^−4^ for *klf4*-overexpression; *t*-test, *p* = 2.93 × 10^−5^) at 24 hpf (Fig 4B). We also analyzed whether the density of *col1a1a*^*+*^ differentiated keratinocytes was affected by knockdown of *klf4* expression and found that *col1a1a*^*+*^ cell density was significantly reduced in *klf4* morphants compared to control embryos (S3 Fig).

Co-injection with *klf4-7mm* mRNA completely rescued the cell densities of *foxi3a*^+^ expressing ionocytes in 24 hpf morphants, while co-injection with *LacZ* mRNA had no such effect (S4A Fig). In addition, Klf4 protein was scarcely detected by immunofluorescence in bud embryos injected with *klf4* MOs as compared with control embryos (S4B Fig). Immunofluorescence with antibodies against Na^+^, K^+^-ATPase or H^+^-ATPase further confirmed that *klf4* knockdown significantly reduced the densities of NaR and HR cells in a dose-dependent manner at 72 hpf, as compared to uninjected wild types or embryos injected with control MOs (S5 Fig). These results demonstrate that Klf4 affects the differentiation of NaR and HR ionocytes as well as *col1a1a*^*+*^ keratinocytes by regulating cell densities of their progenitors.

### Zebrafish Klf4 regulates the proliferation of p63^+^ stem cells by modulating expression of *p53* and *cdkn1a/p21*

**Fig 5:**
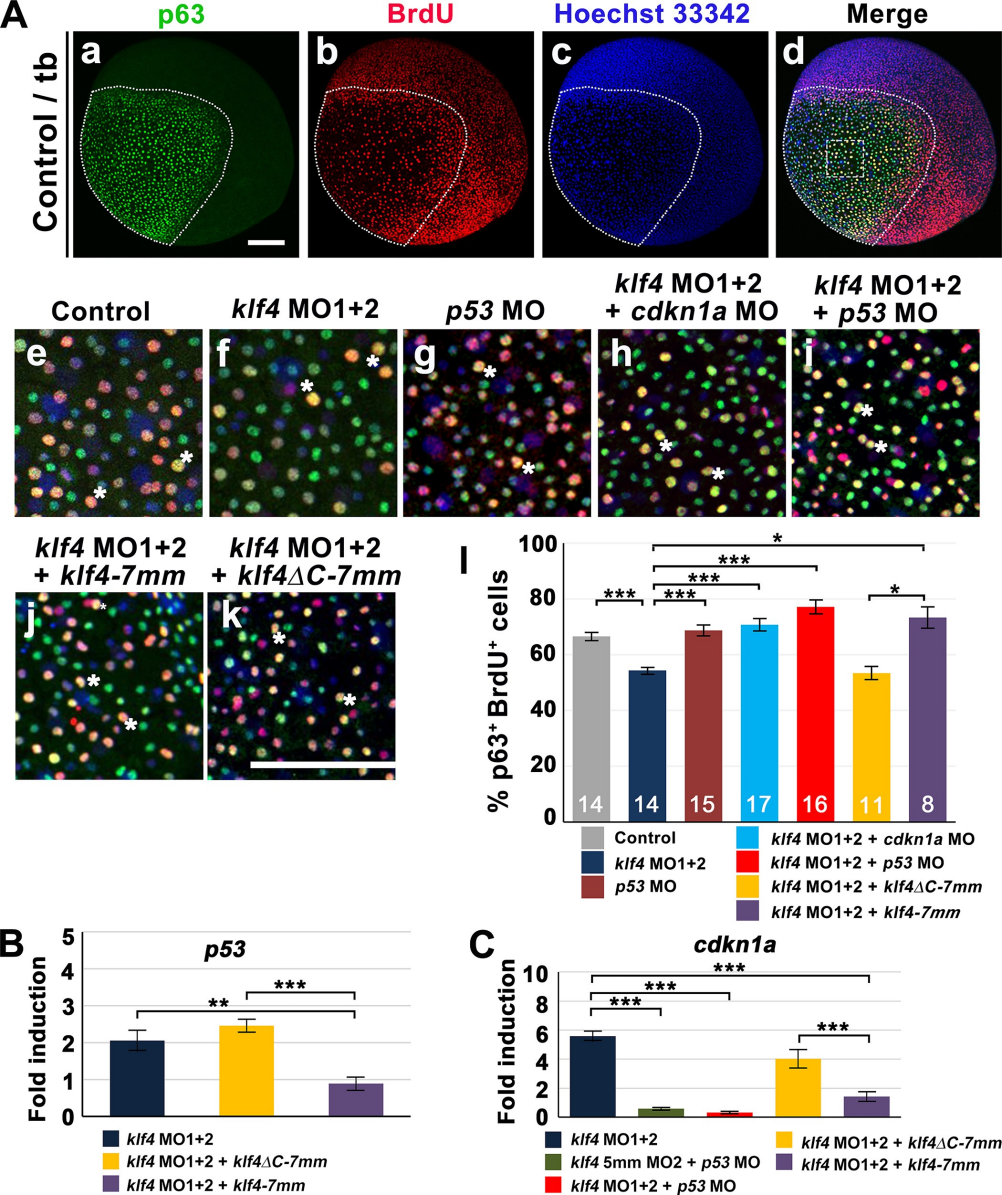
Klf4 maintains epidermal stem cell proliferation by repression of *p53* expression. **(A)** BrdU and p63 labeling were performed on embryos injected with *klf4* 5mmMO2 (control) (a-e), combined *klf4* MO1/*klf4* MO2 (f), *p53* MO (g), combined *cdkn1a* MO/ *klf4* MO1/*klf4* MO2 (h), combined *p53* MO/*klf4* MO1/*klf4* MO2 (i), combined *klf4-7mm* mRNA/*klf4* MO1/*klf4* MO2 (j), or combined *klf4ΔC-7mm* mRNA/*klf4* MO1/*klf4* MO2 (k). Nuclei are counterstained with Hoechst 33342 (c). Both p63^+^ and p63^+^BrdU^+^ cell number were enumerated in the circled areas. Examples of p63 and BrdU colocalized cells (asterisk) are shown. The percentage of p63^+^BrdU^+^ cells in embryos after indicated treatments was quantified (l). (**B**) *p53* expression in embryos injected with combined *klf4* MO1/*klf4* MO2, combined *klf4-7mm* mRNA/*klf4* MO1/*klf4* MO2, or combined *klf4ΔC-7mm* mRNA/*klf4* MO1/*klf4* MO2 was measured by RT-qPCR. (**C**) *cdkn1a*/*p21* expression in embryos after indicated treatments was measured by RT-qPCR. Statistical significance was determined by Student’s *t*-test. *p < 0.05; **p < 0.01; ***p < 0.001. Scale bar, 50 μm. Error bars indicate standard error.

Because *klf4* deficiency resulted in reduced p63^+^ stem cell proliferation rate, we next explored the mechanism through which Klf4 modulates p63^+^ epidermal stem cell proliferation. A reduction in the percentage of p63^+^BrdU^+^ epidermal stem cells was detected in *klf4* bud morphants compared to control embryos (66.52 ± 1.43% vs. 54.36 ± 1.04%; *t*-test, *p* = 2.71 x 10^−7^) (Fig 5Aa-f,l). Furthermore, co-injection of *klf4-7mm* mRNA, but not *klf4* lacking a C-terminal DNA binding domain (*klf4ΔC-7mm*), completely restored the proportion of p63^+^ BrdU^+^ epidermal stem cells to a control level, indicating that reduced p63^+^ epidermal stem cell proliferation is due to *klf4* deficiency (Fig 5Aj-l). Because p53 is known to regulate the G1/S cell cycle checkpoint by transactivation of *CDKN1A/p21* expression [29], and mammalian KLF4 was shown to repress transcription of *p53*, we evaluated *p53* and *cdkn1a/p21* expression in *klf4* morphants [30]. Upregulated expression levels of *p53* and *cdkn1a/ p21* were found by RT-qPCR in *klf4* morphants compared to control embryos at the 5s stage. Upregulation of *p53* and *cdkn1a/p21* was prevented in 5s embryos co-injected with *klf4-7mm* but not *klf4ΔC-7mm* mRNA, demonstrating that upregulation of *p53* and *cdkn1a/p21* is dependent on decreased *klf4* expression (Fig 5B and 5C). A lack of *cdkn1a/p21* upregulation was further observed in 5s embryos co-injected with *klf4* MOs and *p53* MO (Fig 5C). The decreased percentage of p63^+^ BrdU^+^ epidermal stem cells was also completely rescued in embryos co-injected with *klf4* MOs and *p53* MO or *cdkn1a* MO (Fig 5Ah,i,l). Injection of *p53* MO or *cdkn1a* MO also restored p63^+^ epidermal stem cell number and percentage of p63^+^ BrdU^+^ epidermal stem cells in *klf4*^*d5i1*^ heterozygous mutant embryos to levels comparable to *klf4*^+/+^ sibling wild types at bud stage (S6 Fig). In addition, no apoptosis was observed in ventral ectoderm of *klf4* morphants compared to wild-type and control embryos at 5s stage (S7 Fig).

In order to investigate whether Klf4 regulates p63^+^ epidermal stem cell proliferation in a cell-autonomous manner, we produced chimeric embryos by transplanting FITC dextran-labeled wild-type blastomeres into *klf4*-morphant hosts or FITC dextran-labeled *klf4*-morphant blastomeres into wild-type hosts. Chimeric embryos were labeled with BrdU at 80% epiboly and fixed at bud stage. Immunofluorescence was conducted with anti-FITC, anti-p63 and anti-BrdU antibodies. The difference in percentage of FITC^+^BrdU^+^p63^+^ cells in wild-type hosts transplanted with *klf4*-morphant cells compared to *klf4*-morphant hosts transplanted with wild-type blastomeres (S8 Fig) was greater than the difference in percentages of BrdU^+^p63^+^ cells detected in *klf4*-morphant embryos (Fig 5l). This unequal difference between the percentages of FITC^+^BrdU^+^p63^+^ and BrdU^+^p63^+^ cells may be due to variations in wild-type response to morpholino injection. Nevertheless, this result demonstrates that Klf4 cell-autonomously regulates epidermal stem cell proliferation by repressing *p53* expression. Thus, in *klf4*-deficient embryos, p53 activity is not inhibited and activates *cdkn1a/p21* expression to prevent cell cycle progression.

### Klf4 directly binds to the *dlc* promoter to modulate *dlc*-mediated lateral inhibition

**Fig 6:**
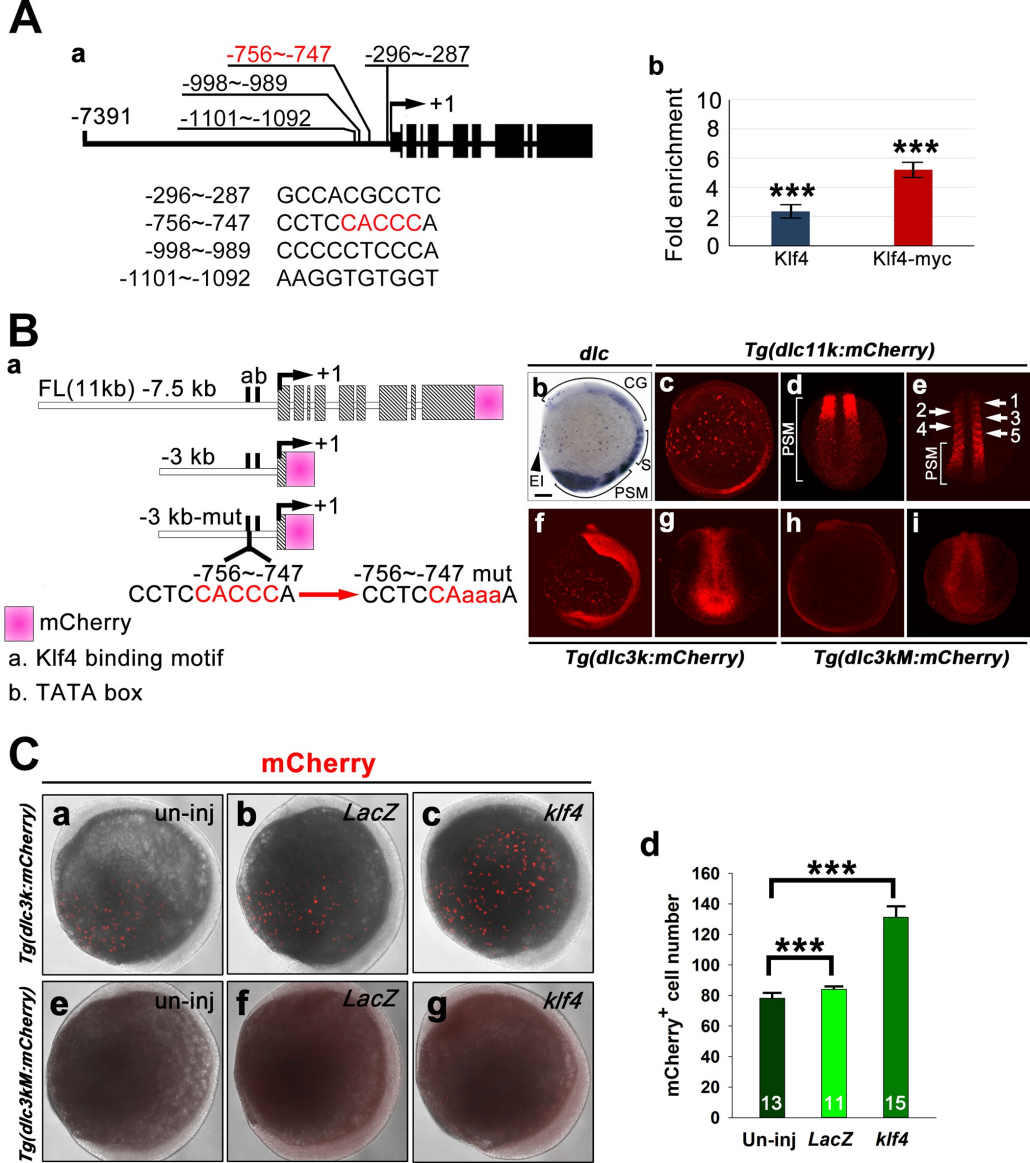
Klf4 directly binds to the *dlc* promoter. (**A**) Four putative KLF-binding motifs were identified in the upstream region of the *dlc* promoter. The core binding motif (CACCC) is indicated by red text (a). Chromatin immunoprecipitation was performed using anti-Klf4 or anti-Myc antibodies, and qPCR of the isolated chromatin revealed significant enrichment of the KLF-binding motif located between -756 to -747 of the *dlc* promoter (b). (**B**) A diagram depicting the organization of *dlc11k-mCherry*, *dlc3k-mCherry*, and *dlc3kM-mCherry* constructs. KLF binding motif is shown in red text, and mutated bases are indicated by lowercase letters (a). Images of 5s embryos labeled with *dlc* antisense RNA probe (b), F1 transgenic embryos from *Tg(dlc11k*:*mCherry)* (c-e), *Tg(dlc3k*:*mCherry)* (f, g) and *Tg(dlc3kM*:*mCherry)* (h, i) are shown. Five somites are indicated (e). (**C**). Uninjected (un-inj) *Tg(dlc3k*:*mCherry)* (a) or *Tg(dlc3kM*:*mCherry)* (e), *LacZ*-injected *Tg(dlc3k*:*mCherry)* (b) or *Tg(dlc3kM*:*mCherry)* (f) and *klf4*-injected *Tg(dlc3k*:*mCherry)*(c) or *Tg(dlc3kM*:*mCherry)* (g) embryos were stained with anti-mCherry antibody at bud stage. Quantification of results from (a-c) is shown (d). Statistical significance was determined by Student’s *t*-test. ***p < 0.001. Error bars indicate standard error. CG, cranial ganglia; EI, epidermal ionocytes; S, somites; PSM, presomitic mesoderm.

The haploinsufficiency of *klf4*^*d5i1*^ was found in p63^+^ stem cells but not in their direct downstream *dlc*^+^ ionocyte progenitors, suggesting there may be additional regulatory mechanisms in *dlc*^+^ cells. To investigate whether Klf4 binds directly to the *dlc* promoter *in vivo*, we used JASPAR, a sparse matrix multiplication benchmark for JAVA/F90/C, to identify four potential KLF binding motifs located in the 5′ upstream region of the *dlc* promoter. We then used Klf4 or Myc antibodies to immunoprecipitate cross-linked chromatin from 5s stage wild-type embryos or embryos injected with *klf4-Myc* mRNA. The KLF binding motif, located at -756 to -747 bp, was significantly enriched in the immunoprecipitated chromatin, as measured by qPCR (Fig 6A).

Moreover, we cloned the entire *dlc* coding gene, including nine exons and eight introns, as well as 7505 bp upstream of the transcription initiation site into a mini-Tol2-mCherry-based vector and established a stable *Tg(dlc11k*:*mCherry*) transgenic line. Expression of mCherry in the epidermal ionocyte domain, cranial ganglia, somites and presomitic mesoderm regions of *Tg(dlc11k*:*mCherry*) F1 embryos recapitulated expression patterns of endogenous *dlc* at the 5s stage (Fig 6Bb-e). To create a mutated KLF binding motif between -756 and -747, we cloned 296 bp of *dlc* exon 1 and 2840 bp upstream of the transcriptional initiation site of the *dlc* promoter containing a wild-type or a mutated KLF binding motif into a mini-Tol2-mCherry-based vector and established two stable transgenic lines, namely wild-type *Tg(dlc3k*:*mCherry)* and mutated *Tg(dlc3kM*:*mCherry)*. mCherry expression was observed in the epidermal ionocyte domain and in nonspecific ectoderm covering the entire trunk of *Tg(dlc3k*:*mCherry)* embryos (Fig 6Bf,g). This observation suggests that the sequence between -7505 and -2840 bp upstream of *dlc* promoter is involved in proper patterning of *dlc* expression in the cranial ganglia, somite and presomitic mesoderm. In addition, more mCherry-expressing cells were detected in the epidermal ionocyte domains of *Tg(dlc3k*:*mCherry)* embryos compared to *Tg(dlc11k*:*mCherry*) embryos. This difference may be attributed to a shorter half-life for full-length Dlc-mCherry fusion protein based on ubiquitination and degradation events. In contrast, no mCherry expression was detected in the epidermal ionocyte domain, and low level mCherry expression was observed in the presomitic mesoderm region of the mutant *Tg(dlc 3kM*:*mCherry)* transgenic line at the 5s stage (Fig 6Bh,i). Furthermore, increased mCherry protein was detected in *klf4*-overexpressing *Tg(dlc3k*:*mCherry)* but not *Tg(dlc3kM*:*mCherry)* transgenic embryos compared to *LacZ*-overexpressing *Tg(dlc3k*:*mCherry)* transgenic embryos at bud stage (Fig 6Ca-g). Taken together, these findings suggest that Klf4 binds directly to the KLF binding motif at -756 to -747 bp to modulate *dlc* transcription.

Mutation of the -756 to -747 upstream KLF binding motif abolished mCherry expression in the ionocyte domain, while knockdown of *klf4* only resulted in decreased cell density of *dlc*^*+*^ ionocyte progenitors at the bud stage (Figs 6Bh and 3Ac,d). These differing observations imply that additional transcription factors may bind to the KLF binding motif or act as essential cofactors for *dlc* expression in the ionocyte expression domain. The transcription factor, Suppressor of Hairless (Su(H)), interacts with the intracellular domain of Notch to activate downstream genes, while Su(H)^DBM^ contains a point mutation in the DNA binding domain and acts as dominant negative to inhibit Notch signaling [31]. To examine the influence of Notch signaling on *dlc* expression in ionocyte progenitors, we further injected dominant-negative *X-Su(H)*^*DBM*^ RNA into 1-cell zygotes of *Tg(dlc3k*:*mCherry)* or *Tg(dlc3kM*:*mCherry)* transgenic lines and evaluated *mCherry*^+^ or endogenous *dlc*^+^ ionocyte progenitor cell numbers (S9 Fig). We observed significant increases in cell numbers for both *mCherry*^+^ and endogenous *dlc*^+^ ionocyte progenitors in *X-Su(H)*^DBM^ -injected *Tg(dlc3k*:*mcherry)* embryos with Notch inhibition. However, no *mCherry*^+^ ionocyte progenitors were detected in *X-Su(H)*^DBM^ RNA-injected *Tg(dlc3kM*:*mCherry)* embryos, despite the increased number of endogenous *dlc*^+^ ionocyte progenitors. These results further demonstrate that the -756 to -747 KLF binding motif on *dlc* promoter is essential for lateral inhibition, and this motif might be additionally regulated by unknown transcription factor(s) that act downstream of *Su(H)*.

In order to further investigate whether Klf4 acts as an activator or suppressor of *dlc* expression, we generated two chimeric constructs, which contained either a VP16 activator or an Engrailed repressor domain linked to a NLS sequence and Klf4 zinc finger DNA binding domain. At the 5s stage, similar *foxi3a*^*+*^ ionocyte cell densities were detected in embryos injected with 50 pg *VP16-klf4* or 130 pg *LacZ* mRNA compared to embryos injected with 130 pg *klf4* mRNA, suggesting that Klf4 is not likely to function as activator (S10H Fig). However, abnormal embryonic development and decreased *foxi3a*^*+*^ ionocyte cell density were identified in embryos injected with 50 pg *engrailed-klf4* mRNA (S11 Fig). Based on preliminary tests of different doses, we injected a very low amount (0.1 pg) of *engrailed-klf4* mRNA and observed a significant increase in *foxi3a*^+^ ionocyte cell density at the 5s stage compared to *LacZ*-overexpressing embryos, which was similar to that seen in *klf4*-overexpressing embryos (S10D Fig). Together, these results suggest that Klf4 functions as repressor of *dlc* expression.

### Zebrafish Klf4 maintains the ionocyte progenitor population by modulating Dlc-mediated lateral inhibition

Klf4 modulation of *dlc*^+^ ionocyte progenitor cell number may be regulated by direct binding of Klf4 to the *dlc* promoter (Fig 6). Several potential explanations may account for the alteration of *dlc*^*+*^ ionocyte progenitor number that resulted from perturbation of *klf4* expression. The first potential explanation is that the cell size may be altered. To examine this possibility, we compared cell diameters after making two assumptions: (1) the ionocyte domain is a two-dimensional single cell layer, and (2) *dlc* expression levels do not change cell size. We compared the normalized *dlc*^*+*^ cell diameters of *klf4* morphants and *klf4*-overexpressing embryos (S12A Fig). An approximately 5.2% larger cell diameter was measured in *klf4* morphants compared to control embryos, leading us to estimate that 9.7% fewer cells should be found in the ionocyte domain. On the contrary, in *klf4*-overexpressing embryos, a 7.9% smaller cell diameter was observed, suggesting that 17.9% more cells should be contained in the ionocyte domain. However, the estimated cell density differences do not quantitatively match the observed reductions in *dlc*^*+*^ ionocyte progenitor numbers (Fig 2Cc). The second possibility is that the output densities of lateral inhibition were changed by perturbation of *klf4* expression. The average normalized nearest spacing between *dlc*^*+*^ cells ranged from 1.29 to 1.54 cell diameters in both *klf4* morphants and *klf4*-overexpressing embryos, which is close to that found in *in vitro* synthetic lateral inhibition circuits [32]. This observation suggests that the range of ionocyte lateral inhibition is relatively short in comparison to *Drosophila* SOP [11, 33]. Furthermore, the normalized nearest spacing between *dlc*^*+*^ cells is not different between *klf4* morphants and *klf4*-overexpressing embryos (S12B Fig). Thus, the output densities of lateral inhibition seem to be unaffected by *klf4* knockdown or overexpression. The third possibility is that Klf4 controls the range in which precursor cells participate in lateral inhibition. The angle between two vectors that extend from the embryo centroid as the vertex to two points on the embryo edges which flank the *dlc*^*+*^ cell domain was measured [10]. This measurement is proportional to the total area of the ionocyte domain, and was 12.1% smaller in *klf4* morphants and 36.3% larger in *klf4*-overexpressing embryos compared to controls (S12C Fig).

We anticipate that a combination of the effects on cell diameter (S12A Fig) and domain size (S12C Fig) is required to account for the experimentally determined differences in cell number that are presented in Fig 2Cc and Fig 3A. True quantitative comparisons are not possible due to the variation of embryo batches and sensitivity of *in situ* detection methods. However, future studies with live time-lapse analysis may be sufficient to fully describe the morphology of alterations induced by perturbation of *klf4* expression. Nevertheless, we uncovered multiple routes by which Klf4 modulates ionocyte development, including controlling proliferation rates of epidermal stem cells, modulating precursor cell numbers prior to lateral inhibition, and influencing the range of ionocyte domain.

In addition to the three possibilities discussed above, we observed some large *dlc*^*+*^ cell clusters in *klf4* overexpressing embryos that were not observed in *klf4* mutant or morphants. When we analyzed the clustering effect in *klf4* morphants and *klf4*-overexpressing embryos, there was no difference in the percentage of *dlc*^*+*^ connected pairs between *klf4* morphants and control embryos, however, *klf4*-overexpressing embryos showed a significantly increased percentage of *dlc*^*+*^ connected pairs (S12D Fig). In both control and *klf4* morphants, the average maximum *dlc*^*+*^ cluster size in an embryo was 2.1 cells, but in *klf4*-overexpressing embryos, the average maximum *dlc*^*+*^ cluster size was significantly increased to 4.1 cells with a highest observed value of 9 cells (S12E and S12F Fig). Furthermore, the *dlc*^*+*^ cell clustering phenotype does not appear to be temporary, because we detected increased *foxi3a*^*+*^cluster size (ranging from 4 to 6 cells) in *klf4*-overexpressing embryos, compared to *LacZ*-overexpressing embryos (2 cells) at 24 hpf (S12G Fig).

Since injection of p*53* or *cdkn1a* MO rescued proliferation of p63^+^ epidermal stem cells in *klf4* morphants at bud stage (Fig 5), we wondered whether injection of *p53* or *cdkn1a* MO could rescue the reduction in differentiated *atp6v1aa*^+^ ionocyte cell density in *klf4* morphants. Although the *p53* MO-mediated rescue of *atp6v1aa*^+^ ionocyte cell density did not reach statistical significance, injection of downstream *cdkn1a* MO did produce a significant rescue effect on *atp6v1aa*^+^ ionocyte cell density of *klf4* morphants at 24 hpf (S13 Fig). This result suggests that *cdkn1a* expression is necessary to produce the reduction in differentiated *atp6v1aa*^+^ ionocyte cell density in *klf4* morphants.

## Discussion

### Klf4 maintains the ionocyte progenitor population by regulating proliferation of epidermal stem cells

In the present study, we uncovered a novel role for Klf4 in zebrafish epidermis development. In zebrafish embryos, *dlc*^+^ ionocyte progenitors are specified and differentiate from epidermal stem cells during late gastrulation [10]. We showed that Klf4 is expressed in p63^+^ epidermal stem cells beginning at 90% epiboly (Fig 1). Knockout or knockdown of *klf4* reduced epidermal stem cell proliferation, resulting in fewer stem cells, which in turn reduced the number of differentiated *dlc*^+^ p63^+^ ionocyte progenitors (Fig 2, S1 Fig). We further demonstrated that zebrafish Klf4 regulates the epidermal stem cell population by repressing *p53* expression. A significant reduction in the percentage of BrdU^+^ epidermal stem cells was also observed in *klf4* morphants and was accompanied by increased expression levels of *p53* and *cdkn1a/p21*. Co-injection of *klf4* MOs with either *p53* MO or *klf4-7mm* mRNA reversed *cdkn1a/p21* upregulation and restored the percentage of BrdU^+^ epidermal stem cells to control level. Co-injection of *cdkn1a* MO also completely rescued the proportion of BrdU^+^ epidermal stem cells, owing to the fact that *cdkn1a/p21* is an essential downstream target gene of P53 in cell cycle regulation [29]. Similar rescue effects on the percentage of BrdU^+^ p63^+^ epidermal stem cells were detected in *klf4*^*d5i1*^ heterozygous mutant embryos after injection of *p53* MO or *cdkn1a* MO (S6 Fig). In addition, injection of *cdkn1a* MO restored *atp6v1aa*^*+*^ differentiated ionocyte cell density in *klf4* morphants at 24 hpf (S13 Fig), indicating that the decreased *dlc*^*+*^ p63^+^ progenitor cell number and reduced cell density of differentiated ionocytes in *klf4* morphants could be attributed to upregulation of *p53* and *cdkn1a/p21* expression. Maintenance of epidermal stem cell proliferation also requires an intact *klf4* C-terminal DNA binding domain, suggesting that Klf4 may directly suppress *p53* expression (Fig 5). Mammalian KLF4 was previously shown to suppress *p53* expression through direct binding to a specific element within the *p53* promoter. Moreover, this repression of *p53* expression is one feature that transforms KLF4 from a tumor suppressor to an oncogene [24]. Therefore, zebrafish Klf4 may have a conserved function as a suppressor of *p53* expression; further study will be required to analyze potential KLF binding motifs within the zebrafish *p53* promoter.

One of our especially intriguing discoveries, which stands in contrast to previous reports using different models, is that Klf4 maintains the ionocyte progenitor population by regulating epidermal stem cell proliferation [17, 34]. Mammalian KLF4 suppresses keratinocyte proliferation by transcriptional activation of *CDKN1A/p21* expression [35]. Nevertheless, some studies have shown that KLF4 can also facilitate cell proliferation [24, 36, 37]. For example, KLF4 plays an essential role in B cell development and in activation-induced B cell proliferation by regulating Cyclin D2 expression [36]. KLF4 also functions as an oncogene to promote proliferation of breast cancer and bladder cancer cells in the presence of RAS^V12^-Cyclin-D1 signaling or the absence of p21^CIP1^ [24, 37]. Altogether, our findings further support the idea that KLF4 may exert distinct functions to regulate stem cell proliferation in a context-dependent manner.

### Klf4 is a master regulator of cell proliferation-mediated tissue patterning in ionocyte development

The effects of cell proliferation on tissue patterning by lateral inhibition were largely ignored until two recent publications highlighted the issue. First, Akanuma et al. [38] showed that polarized localization of Dlc in developing zebrafish V2 neural progenitor cells determines an asymmetric fate of V2a and V2b daughter cells after cell division. Second, In *Drosophila* notum, Hunter et al. showed that Notch signaling-dependent cell cycle rate contributes to lateral inhibition-mediated microchaete patterning [11]. These findings demonstrated the essential role of the cell cycle in asymmetric fate and lateral inhibition-mediated tissue patterning. Similarly, we discovered that the proliferation rate of *dlc*^+^ cells is lower than that of *dlc*^-^ cells during ionocyte determination. Although the underlying mechanisms of this proliferation rate difference remain unclear, our data suggest that klf4 might be a crucial factor, since our loss-of-function experiments showed closer proliferation rates between the two cell types (Fig 2Cb,c, S1Cb,c Fig).

**Fig 7:**
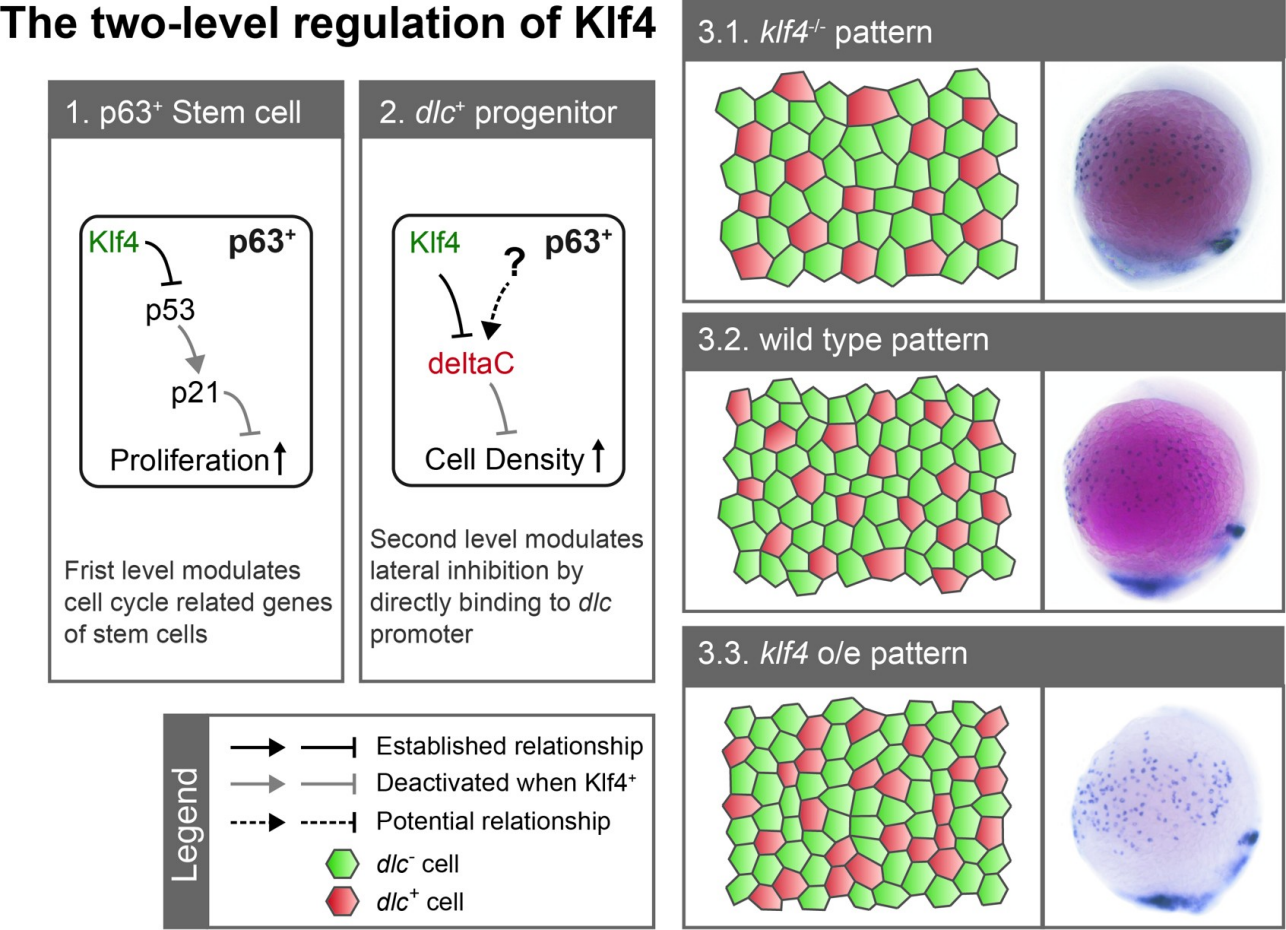
A proposed model for Klf4 functions in maintaining the *dlc*^*+*^ ionocyte progenitor population. Zebrafish Klf4 exerts two-level regulatory activity toward the development of ionocytes. In wild-type embryos, Klf4 represses *p53* expression, preventing the induction of *cdkn1a/p21*, and thereby allowing proper p63^+^ epidermal stem cell proliferation (box 1). At a second level, Klf4 acts as a repressor, regulating Dlc-mediated lateral inhibition by binding to the *dlc* promoter to maintain proper *dlc*^+^ ionocyte progenitor population and patterning during the initiation of ionocyte progenitor differentiation (box 2). The output patterns are illustrated in 2D cell schematics, and representative images of *dlc* mRNA *in situ* hybridization from *klf4* morphants, wild-type and *klf4*-overexpressing embryos at bud stage are shown in box 3. Under *klf4* deficiency, *p53* expression is no longer repressed and *cdkn1a*/*p21* is activated, which results in reduced proliferation of p63^+^ epidermal stem cell and larger/fewer *dlc*^+^ progenitors after selection by lateral inhibition (box 3.1). When *klf4* is overexpressed (box 3.3), more stem cells are selected as ionocyte progenitors and *dlc*^+^ progenitor clusters develop.

In the present study, we describe an important role for Klf4 in regulating epidermal stem cell proliferation and the ionocyte progenitor population, which consequently affects the patterning of ionocytes through *dlc*-mediated lateral inhibition. Thus, we propose a model to describe Klf4 function in the maintenance of the ionocyte progenitor population (Fig 7). In wild-type embryos, Klf4 represses *p53* expression to prevent induction of *cdkn1a/p21*, thereby allowing proper proliferation of p63^+^ epidermal stem cells during late gastrulation. At the same time, Klf4 modulates Dlc-mediated lateral inhibition by repressing *dlc* expression via direct binding to the *dlc* promoter, thus maintaining proper ionocyte progenitor population and patterning. In *klf4*-deficient embryos, *p53* expression is no longer suppressed and *cdkn1a/p21* expression is activated. *cdkn1a/p21* inhibits epidermal stem cell proliferation, and as a consequence, the ionocyte progenitor population is restricted. Conversely, when *klf4* is overexpressed, the ionocyte progenitor population is increased, and an aberrant lateral inhibition pattern is produced by *dlc*^+^ cell clustering. These observations represent novel discoveries in tissue pattern formation by Delta-Notch signaling.

## Materials and methods

### Ethics statement

All animal procedures were approved by the Academia Sinica Institutional Animal Care & Use Committee (AS IACUC) (Protocol ID: 15-12-918). All methods were performed in accordance with the approved guideline.

### Zebrafish strains and maintenance

Zebrafish, including ASAB wild-type, *klf4*^*d5i1*^, *Tg(dlc11k*:*mCherry*)^as33^, *Tg(dlc3k*:*mCherry)*^as34^, and *Tg(dlc3kM*:*mCherry)*^as35^ fish lines, were maintained as previously described [39]. Different stages of embryos were defined according to morphological criteria described previously [40].

### Generation of expression constructs

To generate the expression vector encoding full-length *klf4* with a C-terminal 5x Myc tag (*klf4-Myc*), PCR was conducted using *T7TS-klf4* plasmid DNA as template, and 5′-AACTCGAGATGAGGCAGCCTCCGACT-3′ and 5′-ATTCTAGAGGTAGATGGCGCTT-3′ primers (restriction sites are underlined). PCR product was cloned into pCS2-PMTC2 vector digested with XhoI and XbaI.

To generate *klf4* full-length coding region with seven mismatched nucleotides at the N-terminus (*klf4-7mm*), PCR with 5′-ATGAGaCAaCCgCCaACcGAaTTcGATAGCATGGCACTGAGCGGAA-3′ (mismatched bases are in lowercase) and 5′-TCACTAGTCTATAGATGGCGCTTCATGTG-3′ (restriction site is underlined) primers was conducted, and PCR product was cloned into pGEMT vector. This construct was used as template and 5′-TCACCGGTATGAGaCAaCCgCCaACcGAaTTcGA-3′ and 5′-TCACTAGTC TATAGATGGCGCTTCATGTG-3′ (restriction sites are underlined) primers were used to conduct a second round PCR. PCR product was first digested with AgeI and blunted with Klenow fragment, followed by digestion with SpeI. Digested PCR product was then cloned into a T7TS vector digested with EcoRV and SpeI.

To generate *klf4* lacking DNA binding domain with seven mismatched nucleotides at the N-terminus (*klf4ΔC-7mm*), PCR was conducted using *klf4-7mm* plasmid as template, and 5′-AACTCGAGATGAGACAACCGCCAACCGAA-3′ and 5′-TTTCTAGACTAGTGTGTGGCGATCCGCTT-3′ (restriction sites are underlined) primers. PCR product was cloned into PCS2^+^ vector digested with XhoI and XbaI.

To create the *dlc11k*-*mCherry* plasmid, a 3230 bp long upstream region of the *dlc* gene from -7245 to -4016 bp was amplified by a first PCR using genomic DNA as template and 5′-ATAGGGCCCCATTTGAGAAGAGTGGGACA-3′ and 5′-TCGCCTCACAGTAAGAAAGTCACTGG-3′ (restriction site is underlined) primers. A 4275 bp long upstream region of *dlc* gene from -4030 to +245 bp (+1 corresponding to transcription initiation site) was amplified by a second PCR using genomic DNA as template and 5′-CTTACTGTGAGGCGACAGTGCTAACC-3′ and 5′-TTTCCGCGGCTTTGCCTTCTTGTCTGCTA-3′ primers. A third PCR was conducted to merge these two fragments, which comprise 7505 bp upstream of *dlc* gene. The products of the first and second PCRs were used as templates, and 5′-ATAGGGCCCCATTTGAGAAGAGTGGGACA-3′ and 5′-TTTCCGCGGCTTTGCCTTCTTGTCTGCTA-3′ were used as primers. The PCR product was then cloned into a miniTol2-mCherry vector digested with ApaI and SacII [41, 42]. A 3307 bp *dlc* coding gene region from +219 to +3525 bp was amplified by a fourth PCR using genomic DNA as template and 5′-CGTTCAGTAGCAGACAAGAAGGCAAAG-3′ and 5′-AACTCGAGTACCTGAGGAAGGACAGAA-3′ primers. The final *dlc* 11k gene was combined by PCR using plasmid DNA containing 7.5 kb *dlc* upstream region and the fourth PCR product of 3.3 kb *dlc* coding gene as template, and 5′-ATAGGGCCCCATTTGAGAAGAGTGGGACA-3′ and 5′-AACTCGAGTACCTGAGGAAGGACAGAA-3′ primers. PCR product was then cloned into a miniTol2-mCherry vector digested with ApaI and XhoI.

To build the *dlc3k-mCherry* plasmid, a 3085 bp region upstream of *dlc* gene from -2840 to +245 bp was amplified by PCR using genomic DNA as template and 5′-ATGGGCCCTGCCACTGGATCACACCTCA-3′ and 5′-TTTCCGCGGCTTTGCCTTCTTGTCTGCTA-3′ primers and cloned into a miniTol2-mCherry vector digested with ApaI and SacII.

To create the *dlc3kM-mCherry* plasmid, first and second PCRs were conducted using *dlc3k-mCherry* plasmid DNA as template and respective primer pair 1 (5′-ATGGGCCCTGCCACTGGATCACACCTCA-3′ and 5′-GGCTTT*ttt*TGGAGGGGATTGGCACA-3′) (restriction site is underlined and mutated KLF motif is italicized) and primer pair 2 (5′-CCCTCCA*aaa*AAAGCCCCTCCGCGAT-3′ and 5′-TTTCCGCGGCTTTGCCTTCTTGTCTGCTA-3′). The final PCR was conducted using the first and second PCR products as template, and 5′-ATGGGCCCTGCCACTGGATCACACCTCA-3′ and 5′-TTTCCGCGGCTTTGCCTTCTTGTCTGCTA-3′ primers. The final PCR product was then cloned into a miniTol2-mCherry vector digested with ApaI and SacII.

To generate a chimeric plasmid (*VP16-klf4*), which contains the VP16 activation domain (amino acids 410–490) linked to a Klf4 NLS and Zinc finger DNA binding domain (amino acids 295–396), a first PCR was conducted using *T7TS-klf4* plasmid as template and 5′-CTTGGAATTGACGAGTACGGTGGGGGGTTGCCGGAAGAAT-3′ and 5′-TCTAGATCTAGACTATAGATGGCGCTTCATGTGCAG-3′ (restriction site is underlined) primers. A second PCR was conducted using *pCS2*^*+*^*-NLS VP16AD* plasmid as a template and 5′-GAATTCGAATTCCTGTCGACGGCCCCCCCGAC-3′ and 5′-TTTGGATTCTTCCGGCAACCCCCCACCGTACTCGTCAATTCC-3′ primers. The final PCR was conducted using 1^st^ and 2^nd^ PCR product as template, and 5′-GAATTCGAATTCCTGTCGACGGCCCCCCCGAC-3′ and 5′-TCTAGATCTAGACTATAGATGGCGCTTCATGTGCAG-3′ primers. The final PCR product was then cloned into PCS2+ vector digested with EcoRI and XbaI.

To produce a chimeric plasmid (*engrailed-klf4*), which contains the engrailed repressor domain (amino acids 1–298) linked to Klf4 NLS and Zinc finger DNA binding domain, a first PCR was conducted using *T7TS-klf4* plasmid as template and 5′- CAGAGAAATCTGCTCTGGGATCCGGGTTGCCGGAAGAATCC-3′ and 5′-TCTAGATCTAGACTATAGATGGCGCTTCATGTGCAG-3′ (restriction site is underlined) primers. A second PCR was conducted using *dENG-hoxa1a* plasmid as template and 5′-GAATTCGAATTCATGGCCCTGGAGGATCGCTGCAG-3′ and 5′-TTTGGATTCTTCCGGCAACCCGGATCCCAGAGCAGATTTCTC-3′ primers. The final PCR was conducted using 1^st^ and 2^nd^ PCR product as template and 5′-GAATTCGAATTCATGGCCCTGGAGGATCGCTGCAG-3′ and 5′-TCTAGATCTAGACTATAGATGGCGCTTCATGTGCAG-3′ primers. The final PCR product was then cloned into PCS2^+^ vector digested with EcoRI and XbaI.

### Antisense morpholino oligonucleotide-mediated knockdown

Two translational morpholino oligonucleotides (MOs) previously designed to inhibit Klf4 protein synthesis were used [26]. The MO sequences were as follows: *klf4* MO1: CATGAGTGGAAGGAACGCAAAAG; *klf4* MO2: CAAACTCAGTCGGAGGCTGCCTCAT. The following two control MOs were designed: *klf4* 5mmMO1: CATGAcTGcAAGcAACcgAAAAG, and *klf4* 5mmMO2: CAAA gTCAcTCGcAGGCTGgCTgAT. A total of 1.5 or 3 ng each of *klf4* MO1 and *klf4* MO2, 3 ng each of *klf4* 5mmMO1 and *klf4* 5mmMO2, or 6 ng of *klf4* 5mmMO2 were diluted with Danieau solution, and microinjected into the cytoplasm of 1-2-cell zygotes using an IM300 microinjector (Narishige, Tokyo, Japan). The sequence of *p53* MO and *cdkn1a* MO was as described previously [43, 44].

### Generation of *klf4* mutants using CRISPR-Cas9 system

*klf4* mutant was generated using a CRISPR-Cas9 system. CCTop was used to design four sgRNAs targeting exon 4 [45]. Aligned complementary oligomers of individual sgRNA was cloned into BsmBI-digested pT7-gRNA [46]. sgRNA was synthesized using BamHI-linearized pT7-gRNA and MEGAshortscript T7 Transcription Kit (Ambion, Austin, TX, USA). *klf4* sgRNA (250 pg) and Cas9 protein (500 ng; Tools, Taipei, Taiwan) were co-injected into 1-cell zygotes. Genomic DNA was isolated from pools of 10 injected embryos at 24 hpf. PCR was conducted using forward (5′-CGGCAGCCAGAAGAGAGAATAATGTC-3′) and reverse (5′-TTAACACTACAACCGTCTCACTCAAATGC-3′) primers, and amplified DNA was digested with T7 endonuclease I (T7E1) to evaluate deletion and insertion (indel) efficiency. Only one out of four sgRNAs showed high indel efficiency and the injected embryos were reared to adulthood. Injected fish were designated as the F0 generation. To detect the DNA sequence alterations induced by *klf4* sgRNA, genomic DNA was isolated from clipped tail fin of adult F1 fish, T7E1 digestion was performed and DNA sequencing was conducted to determine whether F1 adult fish carried DNA sequence alterations. *klf4*^*d5i1*^ F1 mutants containing a 5 bp deletion and 1 bp insertion in the sgRNA target site were crossed with wild-type fish to produce the F2 generation. A pair of primers (forward: 5′-GCTCATTTCCCCAGCCGAGG-3′ and reverse: 5′-GTGTGTCCTGTGGTGGGCTTTCA-3′) were used for genotyping of F3 heterozygous or homozygous mutant embryos. Since *klf4*^*d5i1*^ homozygous embryos are viable, F4 homozygous adults were also maintained.

### Whole-mount *in situ* hybridization, double immunofluorescence and fluorescence *in situ* hybridization, whole-mount immunofluorescence, and double immunofluorescence

Whole-mount *in situ* hybridization was conducted on embryos treated with 0.003% phenylthiocarbamide, using digoxigenin-labeled antisense RNA probes and alkaline phosphatase-conjugated anti-digoxigenin antibodies as previously described [47]. T7 RNA polymerase (Thermal Fisher Scientific, Ambion Inc., Waltham, USA) was used to synthesize antisense RNA probes, using EcoRI- linearized *foxi3a* plasmid as a template. SP6 RNA polymerase (Roche, Mannheim, Germany) was used to synthesize antisense RNA probes, using NcoI-linearized *atp1a1a*.*1*, ApaI-linearized *atp6v1aa*, NcoI-linearized *dlc*, NcoI-linearized *foxi3b*, BamHI-linearized *mCherry*, or NcoI-linearized *col1a1a*.

Whole mount immunofluorescence for Klf4 protein and fluorescence *in situ* hybridization for *dlc* mRNA was conducted on 3% H_2_O_2_ permeable embryos. Whole-mount *in situ* hybridization using a digoxigenin-labeled *dlc* RNA probe was conducted first at 60°C. After hybridization wash, embryos were blocked with 1% blocking reagent for 1 h before incubation with rabbit anti-Klf4 antibody (1:50) diluted in 1% blocking reagent at 4°C overnight. After PBST (PBS + 0.1% tween 20) washes for 10 min four times, embryos were incubated with anti-rabbit Alexa-488 (1:200, Thermal Fisher Scientific) at room temperature for 3 h. Embryos were then washed with PBST and blocked with 2% blocking reagent for 1 h before incubation with anti-Digoxigenin-POD (1:500, Roche) diluted in 2% blocking reagent at 4°C overnight. After PBST washes, embryos were incubated with TSA-Cy3 (1:50, Perkin Elmer) diluted in Amplification buffer at 28°C for 1 h. Embryos were then washed with PBST, post fixation with 4% paraformaldehyde for 20 min, PBST washes and stored in 80% glycerol at 4°C.

For labeling epidermal NaR and HR cells, 72 hpf-embryos were fixed with 4% paraformaldehyde at room temperature for 3 to 4 h. After two washes with solution (PBS + 0.1% triton X-100) for 5 min each time, embryos were permeabilized with 100% ice-cold acetone at -20°C for 7 min. Embryos were then washed with dH_2_O and PBST several times, after which they were blocked with 10% serum for 1 h. Embryos were incubated with α5 monoclonal antibody against Na^+^-K^+^-ATPase (1:200, Developmental Studies Hybridoma Bank, Iowa, USA) or a polyclonal antibody against killifish H^+^-ATPase (1:200) [48] diluted with 10% serum at 4°C overnight. After PBST washes, embryos then treated with anti-mouse Alexa 488 antibody (1:200) or anti-rabbit Alexa 568 antibody (1:200, Thermal Fisher Scientific) diluted in 10% serum at room temperature for 3 h. Embryos were washed with PBST and stored in 80% glycerol at 4°C.

Double immunofluorescence for Klf4 and p63 was conducted on embryos fixed with 4% paraformaldehyde for overnight at 4°C. After PBST washes and blocking with 1% blocking reagent (Roche) for 1 h, diluted anti-p63 (1:200, Abcam) antibody and anti-Klf4 (1:50) polyclonal antibody in 1% blocking reagent were added to embryos and incubated at 4°C overnight. Embryos were washed with PBST and incubated with diluted anti-mouse Alexa 488 antibody (1:200) in 0.5% blocking reagent at 4°C overnight. After PBST washes, embryos were incubated with anti-rabbit Alexa 568 antibody (1:200) in 0.5% blocking reagent at 4°C overnight. After PBST washes, embryos were stained with diluted Hoechst 33342 (1:1000, Thermal Fisher Scientific) in PBST for 30 min. After PBST washes, 4% paraformaldehyde fixation, and more PBST washes, embryos were stored in 80% glycerol at 4°C.

Immunofluorescence on chimeric embryos was conducted on fixed BrdU-exposed bud embryos that had been stored in 100% methanol at -20°C. After rehydration and PBST washes, embryos were blocked with 2% blocking reagent for 1 h at RT. Embryos were then incubated with anti-fluorescein-POD in 2% blocking reagent (1:500) at 4°C overnight. After several PBST washes and a rinse with Plus Amplification Diluent (Perkin Elmer), embryos were then incubated with TSA-fluorescein amplification reagent (1:100–1:150) in Plus Amplification Diluent at 28°C for 1 h. After PBST washes, embryos were incubated in 2N HCl for 20 min and washed with PBST several times. After blocking in 1% blocking reagent for 1 h at RT, embryos were incubated at 4°C overnight with rabbit anti-BrdU antibody (1:200; Abcam) that was diluted in 1% blocking reagent. After several PBST washes, embryos were incubated with anti-rabbit Alexa-647 (1:200; Thermo Fisher) in 0.5% blocking reagent at RT for 5 h. After PBST washes, embryos were incubated in mouse anti-p63 antibody (1:200) diluted in 1% blocking reagent at 4°C for one or two days. After several PBST washes, embryos were incubated in mouse Alexa-568 in 0.5% blocking reagent (1:200; Thermo Fisher) at 4°C overnight. Embryos were then washed with PBST and incubated in Hoechst 33342 in PBST (1:1000) for 30 min at RT. After PBST washes, 4% paraformaldehyde fixation and more PBST washes, embryos were embedded in 1% low-melting agar for confocal imaging.

Immunofluorescence for mCherry in *Tg(dlc11k*:*mCherry*) and *Tg(dlc3k*:*mCherry)* transgenic embryos was conducted on 4% paraformaldehyde fixed and dehydrated embryos. Embryos were rehydrated and washed twice with PBSTx (PBS with 0.1% triton X-100) for 5 min. Embryos were then permeabilized with PBS containing 2% triton X-100 for 30 min at RT, after which the samples were washed twice with PBSTx for 5 min. After blocking in 1% blocking reagent for 1 h, embryos were incubated with rat anti-mCherry antibody (1:50–1:150; Thermo Fisher) diluted in 1% blocking reagent at 4°C overnight. After several PBSTx washes, embryos were incubated with anti-rat Alexa-568 antibody (1:200) diluted in 0.5% blocking reagent at 4°C overnight. Embryos were then washed with PBSTx and fixed with 4% paraformaldehyde.

### RNA synthesis, overexpression, and rescue experiments

Capped mRNA (*klf4*, *klf4-7mm*, *klf4ΔC-7mm*, *klf4-Myc*, *X-Su(H)*^*DBM*^, *VP16-klf4*, *engrailed-klf4* or *LacZ*) was synthesized using either a T7 or SP6 mMESSAGE mMACHINE kit (Thermal Fisher Scientific, Ambion Inc.). To ectopically express *klf4*, *klf4* mRNA (130–150 pg) was injected into 1-cell zygotes, and the same amount of *LacZ* mRNA was injected for comparison. To rescue *klf4* morphants, 1-cell zygotes were co-injected with *klf4*-MO1 and *klf4*-MO2 (3 ng each) together with *klf4-7mm* (50 pg) mRNA. Control embryos were co-injected with *LacZ* mRNA (50 pg) and 3 ng each of *klf4*-MO1 and *klf4*-MO2. To ectopically express *X-Su(H)*^*DBM*^, *X-Su(H)*^*DBM*^ (1000 pg) mRNA was injected into 1-cell zygotes of *Tg(dlc3k*:*mCherry)* or *Tg(dlc3kM*:*mCherry)* lines.

### BrdU labeling, TUNEL, rescue and photography

Dechorionated embryos from 80% epiboly were incubated in egg water containing 10 mM BrdU and 15% DMSO for 20 min on ice and washed with egg water. BrdU treated embryos were allowed to grow to bud stage at 28°C before fixation with 4% paraformaldehyde at 4°C overnight. After washing with PBST, embryos were dehydrated through a methanol series and stored in 100% methanol at -20°C overnight. Embryos were incubated with 3% H_2_O_2_ in methanol for 30 min, rehydrated with a methanol series and washed with PBST. Whole-mount *in situ* hybridization using digoxigenin-labeled *dlc* antisense RNA was conducted first at 60 or 65°C. After hybridization washes, embryos were blocked with 2% blocking reagent at room temperature for 1 h before incubation with anti-digoxigenin-POD antibody (1:500, Roche) diluted in 2% blocking reagent at 4°C overnight. After PBST washes, embryos were incubated with TSA-Cy3 (1:50, Perkin Elmer) diluted in Amplification buffer at 28°C for 1 h. Once the reaction was completed, embryos were washed with PBST and incubated in 2N HCl for 20 min. Following PBST washes and blocking in 1% blocking reagent at room temperature for 1 h, embryos were treated with diluted anti-rabbit BrdU antibody (1:200, Abcam) and anti-mouse P63 antibody (1:200) diluted in 1% blocking reagent at 4°C overnight. Embryos were then washed with PBST and blocked in 1% blocking reagent at room temperature for 1 h before incubation with anti-rabbit Alexa-647 antibody (1:200, Thermal Fisher Scientific) and anti-mouse Alexa-488 antibody (1:200) diluted in 0.5% blocking reagent at room temperature for 5 h. After PBST washes, cell nuclei were stained with Hoechst 33342 (1:1000) in PBST for 30 min. Embryos were then washed with PBST, fixed with 4% paraformaldehyde, more PBST washes, and stored in 80% glycerol at 4°C. Rescue experiments were conducted by co-injection of 3 ng each of *klf4*-MO1 and MO2 with 50 pg *klf4-7mm* mRNA, 50 pg *klf4ΔC-7mm* mRNA, 9–12 ng of *p53* MO or *cdkn1a* MO into 1–2 cell zygotes and embryos were allowed to develop to 80% epiboly stage before BrdU incubation.

TUNEL staining was performed as described by the manufacturer’s protocol (Roche) with the following modifications. Embryos were permeabilized with 100% acetone at -20°C for 7 min. Embryos were incubated with alkaline phosphatase-conjugated anti-fluorescein antibody (1:5000) at 4°C overnight. After washing with PBST followed by NTMT solution (100 mM Tris-HCl, pH 9.5, 100 mM NaCl, 50 mM MgCl_2_, 0.1% Tween 20), embryos were stained with NBT/BCIP in NTMT solution. Embryos were then stored in 80% glycerol.

Images of embryos were taken using an AxioCam HRC camera on a Zeiss Axio Imager M1 microscope equipped with a DIC mode. High resolution fluorescent images were taken using a Leica TCS-SP5-MP confocal microscope (Leica, Wetzlar, Germany).

### Chromatin immunoprecipitation (ChIP) and *dlc* promoter analysis by stable transgenesis

*klf4-Myc* (150 pg)-injected or wild-type 5s stage embryos were dechorionated with pronase (Sigma, Munich, Germany) and washed with 1×PBS containing 1×proteinase inhibitor (Roche), before being fixed with 37% formaldehyde (final concentration of 1%) at room temperature for 15 min. The embryos were then incubated with glycine (final concentration of 125 mM) for 10 min, and subsequently washed three times with ice cold PBS. Embryos were lysed by pipetting up and down in cell lysis buffer (10 mM Tris-HCl, pH 8.0, 10 mM NaCl, 0.5% NP-40 and 1×proteinase inhibitor) on ice for 15 min. After centrifugation, the nuclear pellet was resuspended in nuclei lysis buffer (50 mM Tris-HCl, pH 8.0, 10 mM EDTA, 1% SDS and 1×proteinase inhibitor), and then pipetted up and down on ice for 10 min. Two volumes of IP buffer (16.7 mM Tris-HCl, pH 8.0, 167 mM NaCl, 1.2 mM EDTA, 1.1% Triton X-100, 0.01% SDS, and 1×proteinase inhibitor) were added, and the resulting mixtures were aliquoted into tubes (200 μl per tube); the aliquots were then sonicated using Bioruptor Pico sonicator (diagenode, Seraing, Belgium) with the following protocol: 3 repeats of 30 sec ON and 30 sec OFF for 5 cycles for six times. An average chromatin length of 300 bp was used. After sonication, sonicated lysates were centrifuged at 14 k rpm for 15 min, and supernatants were transferred to 1.5 mL tubes and incubated with 50 μl pre-cleaned Protein A agarose beads (Invitrogen) for 1 h at 4°C to remove nonspecifically-bound proteins. After centrifugation at 5 k rpm for 10 min, 50 μl supernatant was removed and used as input control. The rest of the supernatant was diluted 10-fold with IP dilution buffer, divided into two parts, and then incubated with either anti-Myc (Cell Signaling, Beverly, USA), anti-Klf4 or anti-IgG antibody bound to Protein A agarose beads, at a 1:100 dilution at 4°C overnight. After centrifugation at 1500 rpm for 5 min, beads were washed sequentially for 15 min/buffer with ChIP wash buffer A (20 mM Tris-HCl, pH 8.0, 2 mM EDTA, 1% Triton X-100, 0.1% SDS, 150 mM NaCl), buffer B (20 mM Tris-HCl, pH 8.0, 2 mM EDTA, 1% Triton X-100, 0.1% SDS, 500 mM NaCl) and buffer C (10 mM Tris-HCl, pH 8.0, 1 mM EDTA, 0.5% NP-40, 0.1% sodium deoxycholate, 0.25M LiCl), followed by two washes with TE solution at room temperature. Freshly prepared 200 μl elution buffer containing 0.1 M NaHCO_3_ and 1% SDS was added to beads, and then incubated for 15 min at 65°C. After centrifugation at 14 k rpm for 5 min, the elution step was repeated once more. Reverse cross-linking of eluted DNA and protein was performed by incubation in buffer containing final concentrations of 0.2 M NaCl and 0.2 μg/ μl RNase A (sigma) at 65°Covernight. Proteins were removed by a final 0.2 μg/ μl proteinase K (Roche) digestion at 65°C for overnight. After phenol/chloroform extraction, eluted DNA was precipitated using ethanol, and its concentration was determined using the Picogreen kit (Invitrogen P7589). The following primers were used for qPCR: dlc (-296 to -287) forward primer: 5′-GTTGTGGTTAGCGTGGGTTTCCA-3′; dlc (-296 to -287) reverse primer: 5′-GGGACTTTGGACCCTTCAGTTACG-3′; dlc (-756 to -747) forward primer: 5′-TGCCGGTTTAACGACTCACACG-3′; dlc (-756 to -7477) reverse primer: 5′-CGCGTGCCAAGCAATTCTCTAA-3′; dlc (-998 to -989) forward primer: 5′-CACAAACCAAGATTGCGAAGCG-3′; dlc (-998 to -989) reverse primer: 5′-GGTAAAGAGGGCGAAATGGTGG-3′; dlc (-1101 to -1092) forward primer: 5′-AGAAAGCATGCAAGGTGTGGTGAT-3′; dlc (-1101 to -1092) reverse primer: 5′-CGCTTCGCAATCTTGGTTTGTG-3′; the following primers were used for negative binding controls: dlc-nb forward primer: 5′- TCGTTCTGCTGGCGTGGG T-3′; dlc-nb reverse primer: 5′- TTACGCAACGCATGACCTTTCAG-3′.

To investigate the role of the KLF binding motif within -756 to -747 on *dlc* gene expression, 25 pg of *dlc11k*:*mCherry*, *dlc3k*:*mCherry*, or *dlc3kM*:*mCherry* plasmid and 25 pg of transposase mRNA were injected into 1 cell zygotes. Injected embryos were allowed to grow to adulthood. Positive F0 transgenic fish was screened by expression of mCherry and later crossed with wild type fish to generate F1 generation. F2 embryos of three transgenic fish lines obtained by crossing with wild-type fish were then analyzed for mCherry expression patterns at 5s stage. F2 embryos of *Tg(dlc3kM*:*mCherry)* were genotyping confirmed by sequencing.

### Generating chimeric embryos by transplantation

We produced chimeric embryos by transplantation as described [49]. A 3% solution of fluorescein-conjugated dextran (MW 10,000, Invitrogen) alone or mixed with *klf4* MO1 and MO2 was injected into 1-2-cell zygotes. Approximately 50 to 150 blastomeres from wild-type or both *klf4* MO1 and *klf4* MO2-injected embryos were transplanted into *klf4*-morphant or wild-type hosts at a region above the blastoderm margin at a developmental stage between sphere and 40% epiboly.

### Quantification of cell number and area

The number of stained ionocytes or keratinocytes was determined using ImageJ software as follows: (i) an image was loaded in ImageJ; (ii) ‘Cell counter’ was selected from the ‘Analyze’ item in the Plugins menu; (iii) ‘Initialize’ was selected; (iv) the software output cell number was recorded. The ionocyte/keratinocyte domain areas in the yolk ball of bud embryos and in the yolk ball or yolk extension of 24 hpf embryos were quantified using ImageJ software as follows: (i) a scale bar image of appropriate magnification was loaded in ImageJ; (ii) a line was drawn over the scale bar to determine the conversion factor between pixel number and length; (iii) from the Analyze menu, ‘set scale’ was selected to define parameters, including distance in pixels, known distance, pixel aspect ratio and unit of length; (iv) a ‘polygon symbol’ was used to draw the outline of the yolk ball or yolk extension; (v) from the Analyze menu, ‘measure’ was selected to determine area.

### Statistical methods

Values are presented as mean ± s.e.m. unless otherwise noted. Two-tailed Student’s *t*-test with unequal variance was performed in Microsoft Excel.

## Supporting information

S1 Fig Knockdown of *klf4* reduced proliferation of epidermal stem cells and *dlc*^+^ ionocyte progenitor cell number. **(A, B)** Images of BrdU-labeled embryos injected with *klf4* 5mm MO2, followed by staining with *dlc* antisense RNA, and stained with anti-p63 and anti-BrdU antibodies at bud stage are shown (a-c) Both p63^+^ and p63^+^ BrdU^+^ cell number were enumerated in the circled area of control or *klf4* morphant embryos. Enlarged images of *klf4* 5mm MO2 or combined *klf4* MO1 and *klf4* MO2-injected embryos stained with p63 and BrdU or *dlc* RNA probe and BrdU are shown (d, e). Examples of BrdU colocalization with p63 or *dlc* are indicated by arrowheads, while p63 or *dlc*-expressing cells without BrdU staining are indicated by arrows. (**C**) Quantitative results from (A, B). Total p63^+^ or *dlc*^-^ p63^+^ cell numbers (open bars) with BrdU^+^ cell numbers (filled bar) of control or *klf4* morphant embryos at bud stage are shown in (a) and (b). *dlc*^*+*^p63^+^ or *dlc*^*+*^p63^-^ cell numbers (open bars) with BrdU^+^ cell numbers (filled bar) of control or *klf4* morphant embryos at bud stage are shown in (c) and (d). Statistical significance is indicated for comparisons of total cell numbers (open box) or BrdU^+^ cell numbers (filled box). Individual percentages of p63^+^BrdU^+^, *dlc*^-^ p63^+^ BrdU^+^, *dlc*^+^p63^+^ BrdU^+^ or *dlc*^+^ p63^-^ BrdU^+^ cells of control or *klf4* morphant embryos at bud stage are indicated within the bar. Embryos are shown in lateral view. Statistical significance was determined by Student’s *t*-test. NS, not significant; **p < 0.01; ***p < 0.001. Scale bars, 50 μm. Error bars indicate standard error.
(TIF)

S2 Fig Homozygous *klf4*^*d5i1*^ mutant embryos exhibit reduced cell density of ionocyte progenitors that express *dlc* and *foxi3a*. Images of wild-type (WT) and *klf4*^-/-^ embryos stained with *dlc* antisense RNA at bud stage are shown (**A**, **B**). Quantification of cell densities of *dlc*^*+*^ ionocyte progenitors in yolk balls of wild-type (N = 5, n = 158) and *klf4*^-/-^ (N = 5, n = 117) embryos is shown (**C**). Images of wild-type and *klf4*^-/-^ embryos stained with *foxi3a* antisense RNA at 5s stage are shown (**D**, **E**). Quantification of cell densities of *foxi3a*^*+*^ ionocyte progenitors in yolk balls of wild-type (N = 7, n = 202) and *klf4*^-/-^ (N = 6, n = 166) embryos is shown (**F**). Statistical significance was determined by Student’s *t*-test. *p < 0.05. Scale bars, 200 μm. Error bars indicate standard error.
(TIF)

S3 Fig Knockdown of *klf4* reduced cell density of *col1a1a*-expressing keratinocytes. Embryos were injected with *klf4* 5mm MO2 (**A**) or both *klf4* MO1 and *klf4* MO2 (**B**) and hybridized with *col1a1a* antisense RNA probe at 24 hpf. Quantification of cell density of *col1a1a*^+^ keratinocytes in yolk balls of control and *klf4* morphants is shown (**C**). Statistical significance was determined by Student’s *t*-test. ***p < 0.001. Scale bars, 200 μm. Error bars indicate standard error.
(TIF)

S4 Fig Co-injection of *klf4* mRNA rescues the cell densities of *foxi3a* expressing ionocytes in *klf4* morphants, and specificity of *klf4* MOs. **(A)** Restoration of cell density of *foxi3a*^+^ ionocytes was detected in yolk extensions of embryos co-injected with combined *klf4* MO1/*klf4* MO2/*klf4 -7mm* (c) mRNA, but not with *LacZ* (b) mRNA at 24 hpf. A wild type embryo containing *foxi3a*^+^ ionocytes (a) is shown. Quantification of cell density of *foxi3a*^*+*^ ionocytes in yolk extensions of embryos with indicated treatments are shown (d). Scale bar, 200 μm. (**B**) Klf4 protein was scarcely detected in bud stage embryos injected with both *klf4* MO1 and *klf4* MO2 (d, f) compared to *klf4* 5mmMO2-injected control embryos (a, c). Nuclei are counterstained with Hoechst 33342 (b, e). Lateral views of embryos are shown. Scale bar, 50 μm. Statistical significance was determined by Student’s *t*-test. NS, not significant; *** *p*<0.001. Error bars indicate standard error.
(TIF)

S5 Fig Knockdown of *klf4* decreases cell densities of NaR and HR cells at 72 hpf. (**A**) Na^+^, K^+^-ATPase-rich (NaR) cell density was reduced in yolk balls of embryos injected with different amounts of combined *klf4* MO1 and *klf4* MO2 (c, d), as compared to uninjected wild type (a) and control embryos injected with combined *klf4* 5mmMO1 and *klf4* 5mmMO2 (b). NaR cell density in yolk balls of uninjected wild type, embryos injected with combined *klf4* 5mmMO1 and *klf4* 5mmMO2, or the indicated amounts of combined *klf4* MO1 and *klf4* MO2 are shown (e). (**B**) H^+^-ATPase-rich (HR) cell density was reduced in yolk balls of embryos injected with different amounts of *klf4* MO1 and *klf4* MO2 (c, d), as compared to uninjected wild type (a) and control embryos injected with *klf4* 5mmMO1 and *klf4* 5mmMO2 (b). HR cell density in yolk balls of uninjected wild type, embryos injected with *klf4* 5mmMO1 and *klf4* 5mmMO2, or the indicated amounts of *klf4* MO1 and *klf4* MO2 is shown (e). Embryos are shown in lateral view. Significance was determined by Student’s *t*-test. **p < 0.01, ***p < 0.001. Scale bar, 300 μm. Error bars indicate the standard error.
(TIF)

S6 Fig Co-injection of *p53* or *cdkn1a* MO rescued epidermal stem cell proliferation in heterozygous *klf4*^*d5i1*^ mutant embryos. BrdU and p63 colabeling was performed on *klf4*^*+/+*^ or *klf4*^*+/-*^ embryos that were uninjected **(a-c, f-h**), or injected with *cdkn1a* MO (**d**, **i**) or *p53* MO (**e**, **j**) at bud stage. Examples of p63 and BrdU colocalized (arrowhead) or non colocalzed (arrow) cells are shown. Both p63^+^ and p63^+^BrdU^+^ cell numbers were enumerated in the circled area of embryos under different treatments. Quantification of p63^+^ cell numbers (open bars) or p63^+^BrdU^+^ cell numbers (filled bars) are shown (**k**). Quantification of the percentage of p63^+^BrdU^+^ cells are shown (**l**). Statistical significance was determined by Student’s *t*-test. NS, not significant; *p < 0.05; **p < 0.01; ***p < 0.001. Error bars indicate standard error.
(TIF)

S7 Fig Knockdown of *klf4* does not induce apoptosis. TUNEL staining was not detected in the ventral ectoderm of wild-type (**A**), embryos injected with either *klf4* 5mmMO2 (**B**), or combined *klf4* MO1 and *klf4* MO2 (**C**) at 5s stage.
(TIF)

S8 Fig Klf4 regulates p63 epidermal stem cell proliferation in a cell-autonomous manner. Representative images of chimeric embryos generated by transplantation of fluorescein dextran-labeled wild-type blastomeres into a *klf4*-morphant host (**A**-**E**), or *klf4*-morphant blastomeres into a wild-type host (**F**-**J**). Bud stage embryos were stained with anti-FITC, anti-p63 and anti-BrdU antibodies. Nuclei are counterstained with Hoechst 33342 (E, J). Arrowheads indicate FITC^+^p63^+^BrdU^+^ (A, D, F, I) cells, while arrows indicate FITC^+^p63^+^ (A, D, F, I) cells. Quantification of percentage of FITC^+^p63^+^BrdU^+^ cells in these chimeric embryos is shown (**K**). Student’s *t*-test. ***p < 0.001. Error bars indicate standard error.
(TIF)

S9 Fig Mutation of KLF binding motif within -756 to -747 of the *dlc* promoter in *Tg(dlc3kM*:*mCherry)* embryos results in absent response to Notch signaling. **(A)** Images of *Tg(dlc3k*:*mCherry)* (a-c) and *Tg(dlc3kM*:*mCherry)* (d-f) embryos at bud stage. *dlc* and *mCherry* signals were detected by *in situ* hybridization. (**B**) *X-Su(H)*^*DBM*^-injected *Tg(dlc3k*:*mCherry)* (a-c) or *Tg(dlc3kM*:*mCherry)* (d-f) embryos are shown. Because the *dlc3k* and *dlc3kM* promoter sequences both contain 296 bp 5′ untranslated region of *dlc* mRNA, each fragment can be hybridized with *dlc* RNA probe containing full-length cDNA. Similar patterns of *mCherry*^+^ and *dlc*^*+*^ ionocyte progenitors were found in both transgenic lines. (**C**) *mCherry*^+^ or *dlc*^*+*^ cell numbers were quantified in *Tg(dlc3k*:*mCherry)* or *X-Su(H)*^*DBM*^-injected *Tg(dlc3k*:*mCherry)* embryos (a, b). Quantification of *dlc*^*+*^ cell number in *Tg(dlc3kM*:*mCherry)* or *X-Su(H)*^*DBM*^-injected *Tg(dlc3kM*:*mCherry)* embryos is shown (c). Student’s *t*-test. *p < 0.05; ***p < 0.001. Scale bar, 100 μm. Error bars indicate standard error.
(TIF)

S10 Fig Klf4 functions as a repressor to regulate *dlc* expression in the epidermal ionocyte domain. *LacZ*, *klf4* or *engrailed-klf4*-injected embryos were hybridized with *foxi3a* antisense RNA probe at 5s stage (**A**-**C**). Cell density of *foxi3a*^+^ ionocytes in different treatment groups from one representative experiment is shown in the graph (**D**). Images of *LacZ*, *klf4* or *VP16-klf4*-injected embryos hybridized with *foxi3a* antisense RNA probe at 5s stage are shown (**E**-**G**). Cell density of *foxi3a*^+^ ionocytes in different treatment groups from one representative experiment is shown in the graph (**H**). Student’s *t*-test. NS, not significant; ***p < 0.001. Scale bar, 200 μm. Error bars indicate standard error.
(TIF)

S11 Fig Abnormal embryonic development and reduced *foxi3a*^*+*^ cell density is identified in embryos injected with 50 pg *engrailed-klf4* mRNA. *LacZ*, *klf4* or *engrailed-klf4*-injected embryos were hybridized with *foxi3a* antisense RNA probe at 5s stage (**A**-**D**). Cell density of *foxi3a*^+^ ionocytes in the yolk balls of different treatment groups from one representative experiment is shown in the graph (**E**). Student’s *t*-test. ***p < 0.001. Scale bar, 200 μm. Error bars indicate standard error.
(TIF)

S12 Fig Overexpression of *klf4* increases connected *dlc*^+^ ionocyte progenitor number and reduces distance among adjacent *dlc*^+^ ionocyte progenitors. **(A)** Cell diameter of *dlc*^+^ cells was altered by both loss (green) and gain (red) of *klf4* function. Values were normalized to control (blue), and individual measurements from embryos (n ≥ 20) are depicted in circles. **(B)** Nearest spacing of *dlc*^+^ cells were not altered by perturbing *klf4* expression. The nearest distances between *dlc*^+^ cells in the central area (within 40% of embryo diameter) of each embryo were measured in units defined by the average cell diameter, i.e. real distance divided by cell diameter. The normalization and color coding are identical to (A). **(C)** The range of ionocyte domain was altered, as measured by the angle between two vectors originating at the embryo centroid and extending to the edges of the *dlc*^+^ domain. **(D)** The percentage of connected *dlc*^+^ cell pairs was increased by *klf4* overexpression. A connected pair is defined as the distance between 2 *dlc*^+^ cells being less than 1.25-cell diameters. **(E)** Maximum *dlc*^+^ cells cluster number of embryos is increased by *klf4* overexpression. Cell clusters are defined by number of cells that form contiguous pairs. An isolated cell (nearest distance > 1.25-cell diameter long) is cluster number 1, a paired cell has cluster number 2, A cluster of three has cluster number 3, and so on. **(F)** Representative images from *klf4* overexpression in (E). Three images of different embryos from control and *klf4* mRNA overexpression groups were selected to show the maximum cluster numbers found. Arrowheads indicate *dlc*^+^ cells; # = cluster number. All measurements were made from the same data sets in Fig 3A. (**G**) Representative images show *foxi3a*^+^ cell clusters on the yolk ball in *klf4*-overexpressing embryos at 24 hpf. Embryo heads to the left. Statistical significance was determined by Student’s *t*-test. NS, not significant; *p < 0.05; ***p < 0.001. Error bars indicate standard deviation.
(TIF)

S13 Fig Co-injection of *cdkn1a* MO but not *p53* MO rescues cell density of *atp6v1aa*^+^ ionocytes. Images are shown of yolk extensions from embryos injected with *klf4* 5mm MO2 (**A**), combined *klf4* MO1 and *klf4* MO2 (**B**), combined *p53* MO/ *klf4* MO1/*klf4* MO2 (**C**), or combined *cdkn1a* MO/ *klf4* MO1/*klf4* MO2 (**D**) after hybridization with *atp6v1aa* antisense RNA probe at 24 hpf. Cell density of *atp6v1aa*^+^ ionocytes in the yolk extensions of different treatment groups was quantified (**E**). Student’s *t*-test. NS, not significant; ***p < 0.001. Scale bar, 200 μm. Error bar indicates standard error.
(TIF)
