# Supplementary figures and images for: Zebrafish Klf4 maintains the ionocyte progenitor population by regulating epidermal stem cell proliferation and lateral inhibition
Source: PLoS Genet. 2019 Apr 1;15(4):e1008058. doi: 10.1371/journal.pgen.1008058 (PMC6459544; doi:10.1371/journal.pgen.1008058)

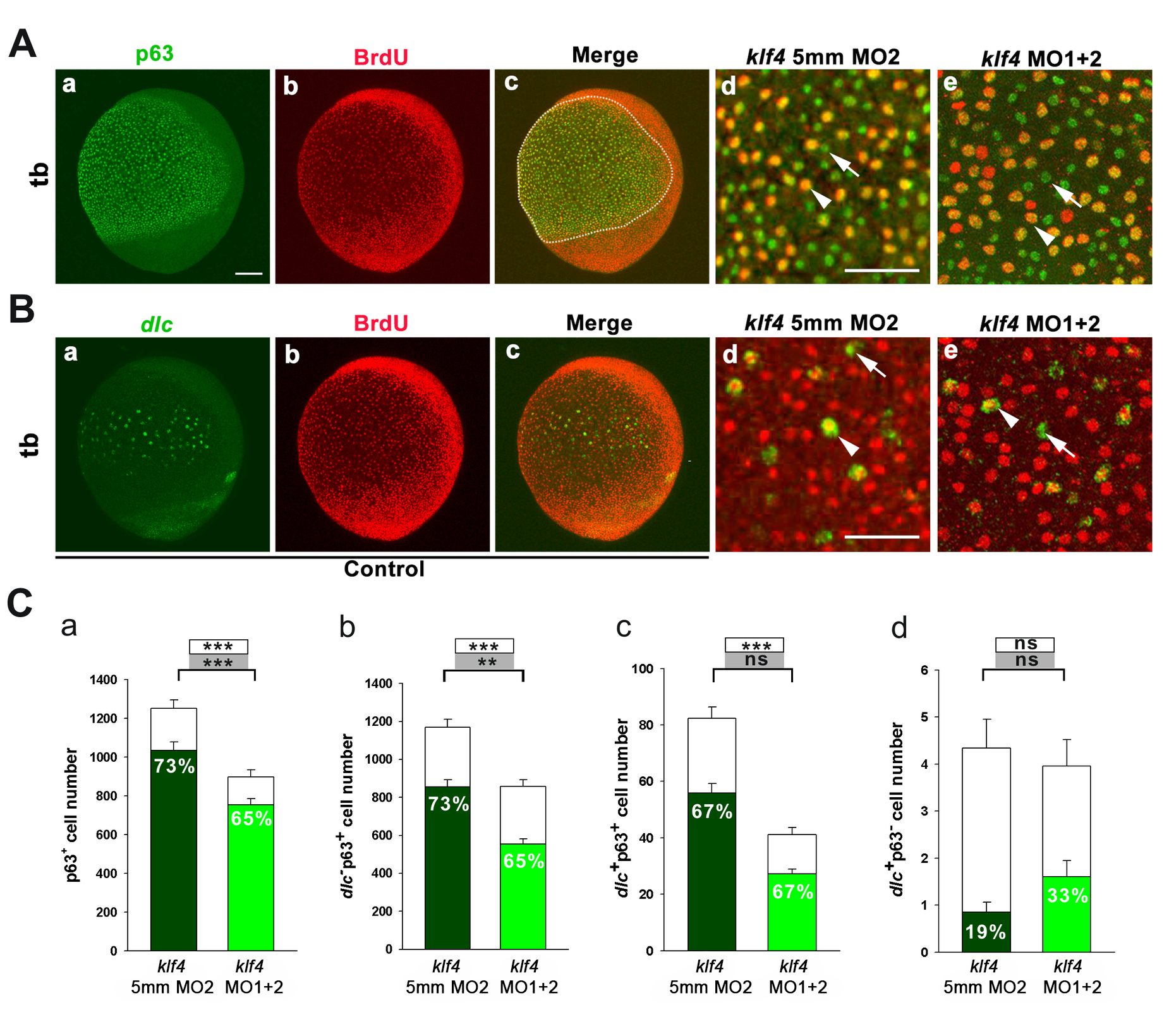

Supplement: S1 Fig — (A, B) Images of BrdU-labeled embryos injected with klf4 5mm MO2, followed by staining with dlc antisense RNA, and stained with anti-p63 and anti-BrdU antibodies at bud stage are shown (a-c) Both p63+ and p63+ BrdU+ cell number were enumerated in the circled area of control or klf4 morphant embryos. Enlarged images of klf4 5mm MO2 or combined klf4 MO1 and klf4 MO2-injected embryos stained with p63 and BrdU or dlc RNA probe and BrdU are shown (d, e). Examples of BrdU colocalization with p63 or dlc are indicated by arrowheads, while p63 or dlc-expressing cells without BrdU staining are indicated by arrows. (C) Quantitative results from (A, B). Total p63+ or dlc- p63+ cell numbers (open bars) with BrdU+ cell numbers (filled bar) of control or klf4 morphant embryos at bud stage are shown in (a) and (b). dlc+p63+ or dlc+p63- cell numbers (open bars) with BrdU+ cell numbers (filled bar) of control or klf4 morphant embryos at bud stage are shown in (c) and (d). Statistical significance is indicated for comparisons of total cell numbers (open box) or BrdU+ cell numbers (filled box). Individual percentages of p63+BrdU+, dlc- p63+ BrdU+, dlc+p63+ BrdU+ or dlc+ p63- BrdU+ cells of control or klf4 morphant embryos at bud stage are indicated within the bar. Embryos are shown in lateral view. Statistical significance was determined by Student’s t-test. NS, not significant; **p < 0.01; ***p < 0.001. Scale bars, 50 μm. Error bars indicate standard error. (TIF) [file pgen.1008058.s001.tif]

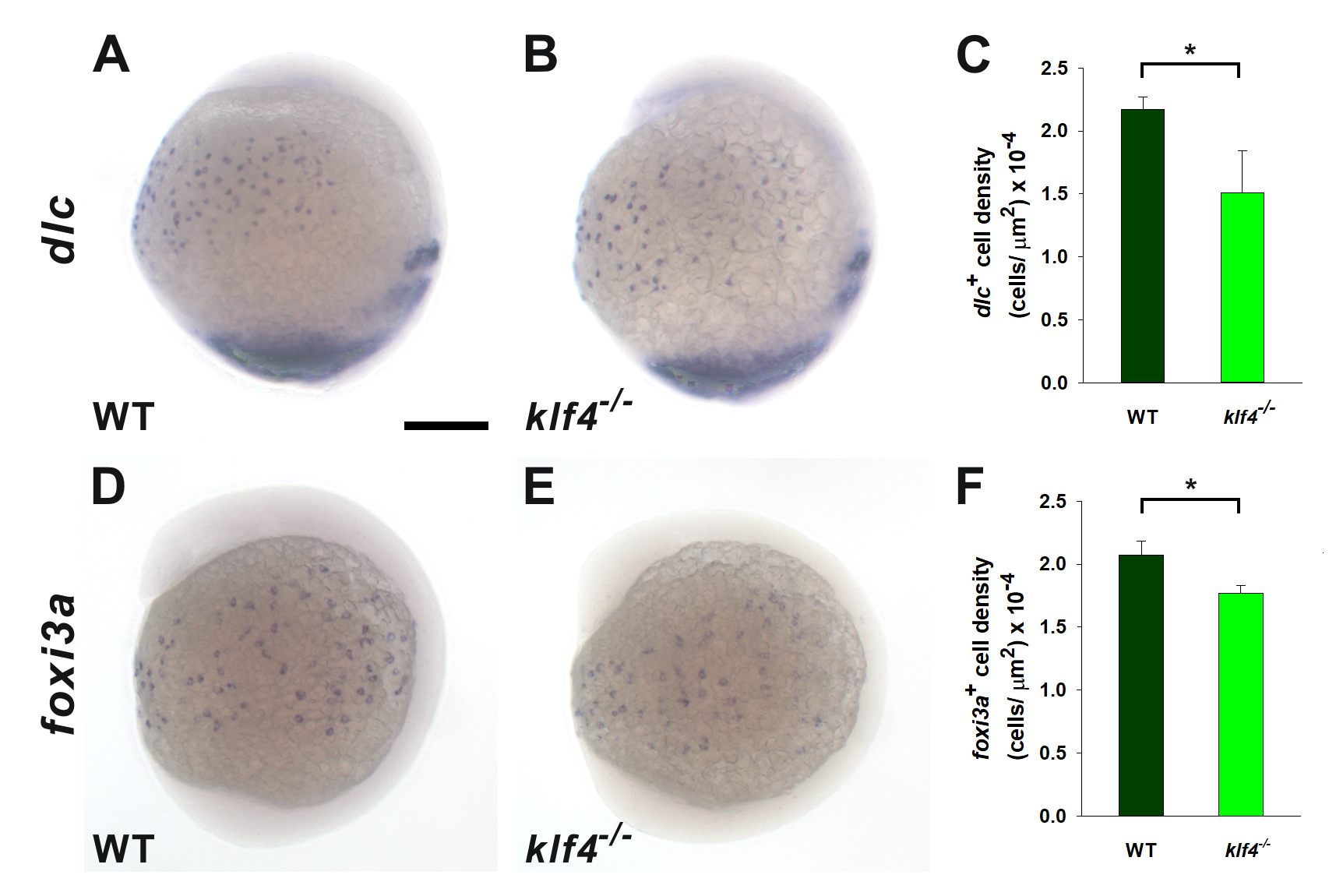

Supplement: S2 Fig — Images of wild-type (WT) and klf4-/- embryos stained with dlc antisense RNA at bud stage are shown (A, B). Quantification of cell densities of dlc+ ionocyte progenitors in yolk balls of wild-type (N = 5, n = 158) and klf4-/- (N = 5, n = 117) embryos is shown (C). Images of wild-type and klf4-/- embryos stained with foxi3a antisense RNA at 5s stage are shown (D, E). Quantification of cell densities of foxi3a+ ionocyte progenitors in yolk balls of wild-type (N = 7, n = 202) and klf4-/- (N = 6, n = 166) embryos is shown (F). Statistical significance was determined by Student’s t-test. *p < 0.05. Scale bars, 200 μm. Error bars indicate standard error. (TIF) [file pgen.1008058.s002.tif]

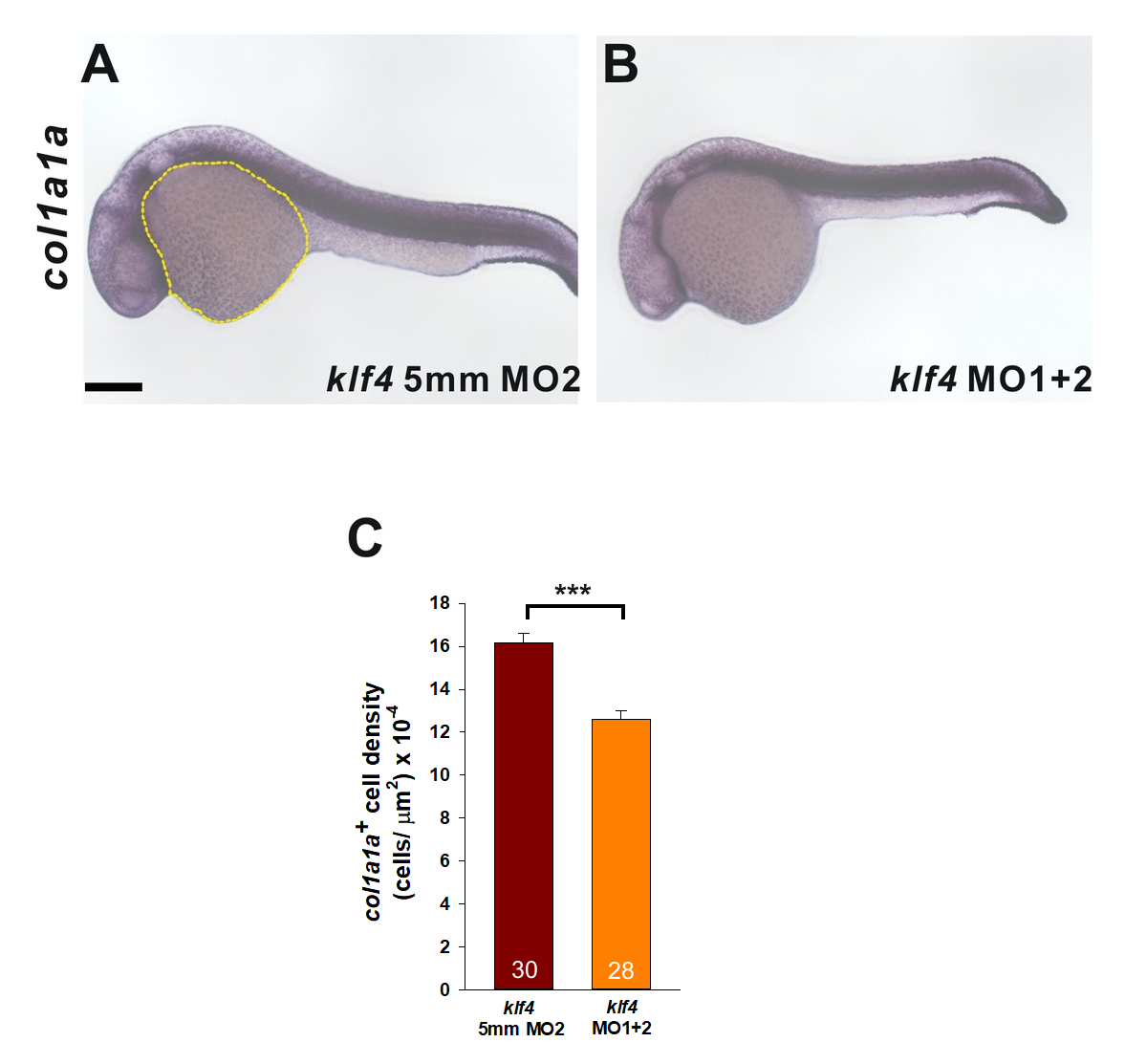

Supplement: S3 Fig — Embryos were injected with klf4 5mm MO2 (A) or both klf4 MO1 and klf4 MO2 (B) and hybridized with col1a1a antisense RNA probe at 24 hpf. Quantification of cell density of col1a1a+ keratinocytes in yolk balls of control and klf4 morphants is shown (C). Statistical significance was determined by Student’s t-test. ***p < 0.001. Scale bars, 200 μm. Error bars indicate standard error. (TIF) [file pgen.1008058.s003.tif]

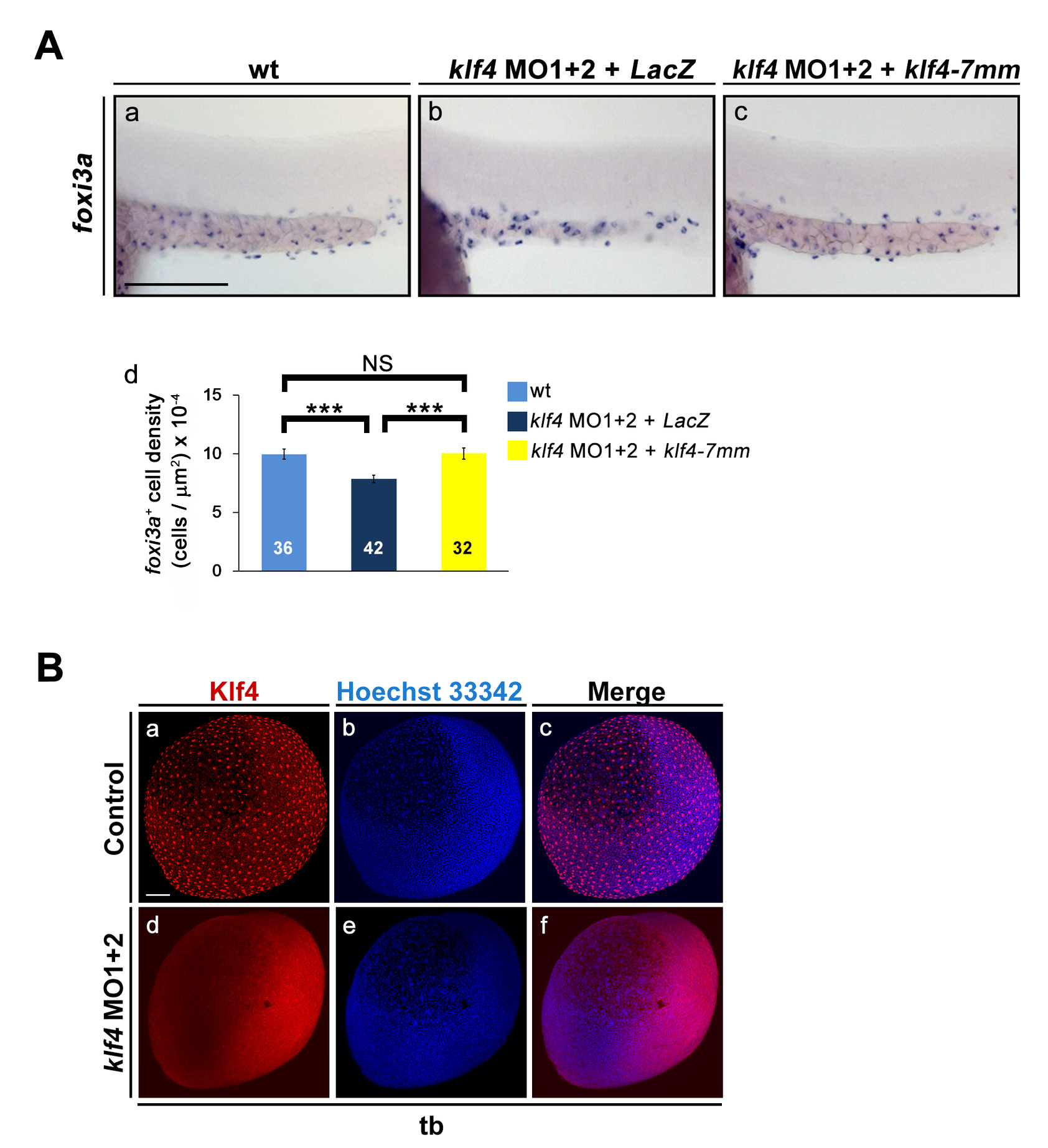

Supplement: S4 Fig — (A) Restoration of cell density of foxi3a+ ionocytes was detected in yolk extensions of embryos co-injected with combined klf4 MO1/klf4 MO2/klf4 -7mm (c) mRNA, but not with LacZ (b) mRNA at 24 hpf. A wild type embryo containing foxi3a+ ionocytes (a) is shown. Quantification of cell density of foxi3a+ ionocytes in yolk extensions of embryos with indicated treatments are shown (d). Scale bar, 200 μm. (B) Klf4 protein was scarcely detected in bud stage embryos injected with both klf4 MO1 and klf4 MO2 (d, f) compared to klf4 5mmMO2-injected control embryos (a, c). Nuclei are counterstained with Hoechst 33342 (b, e). Lateral views of embryos are shown. Scale bar, 50 μm. Statistical significance was determined by Student’s t-test. NS, not significant; *** p<0.001. Error bars indicate standard error. (TIF) [file pgen.1008058.s004.tif]

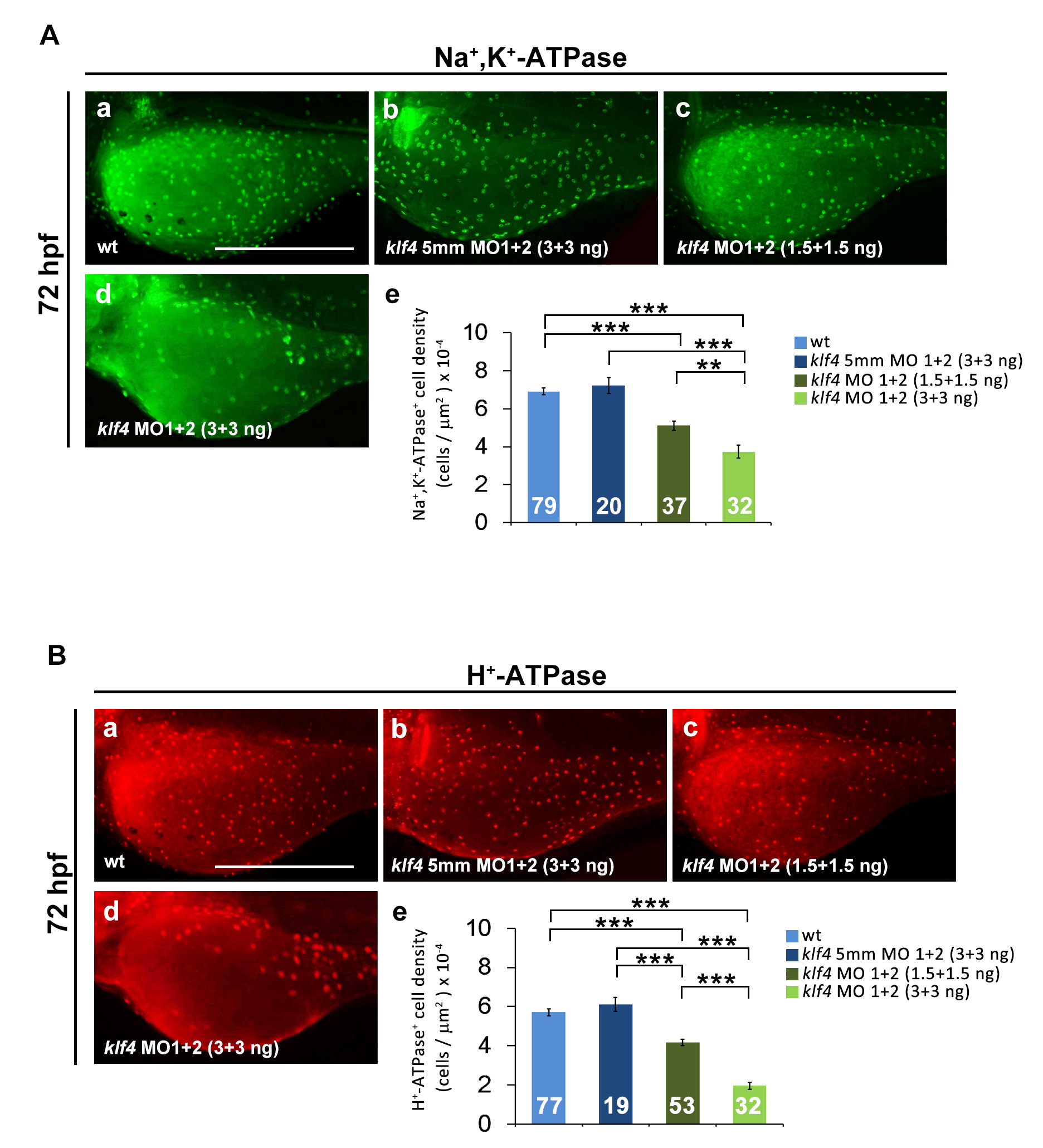

Supplement: S5 Fig — (A) Na+, K+-ATPase-rich (NaR) cell density was reduced in yolk balls of embryos injected with different amounts of combined klf4 MO1 and klf4 MO2 (c, d), as compared to uninjected wild type (a) and control embryos injected with combined klf4 5mmMO1 and klf4 5mmMO2 (b). NaR cell density in yolk balls of uninjected wild type, embryos injected with combined klf4 5mmMO1 and klf4 5mmMO2, or the indicated amounts of combined klf4 MO1 and klf4 MO2 are shown (e). (B) H+-ATPase-rich (HR) cell density was reduced in yolk balls of embryos injected with different amounts of klf4 MO1 and klf4 MO2 (c, d), as compared to uninjected wild type (a) and control embryos injected with klf4 5mmMO1 and klf4 5mmMO2 (b). HR cell density in yolk balls of uninjected wild type, embryos injected with klf4 5mmMO1 and klf4 5mmMO2, or the indicated amounts of klf4 MO1 and klf4 MO2 is shown (e). Embryos are shown in lateral view. Significance was determined by Student’s t-test. **p < 0.01, ***p < 0.001. Scale bar, 300 μm. Error bars indicate the standard error. (TIF) [file pgen.1008058.s005.tif]

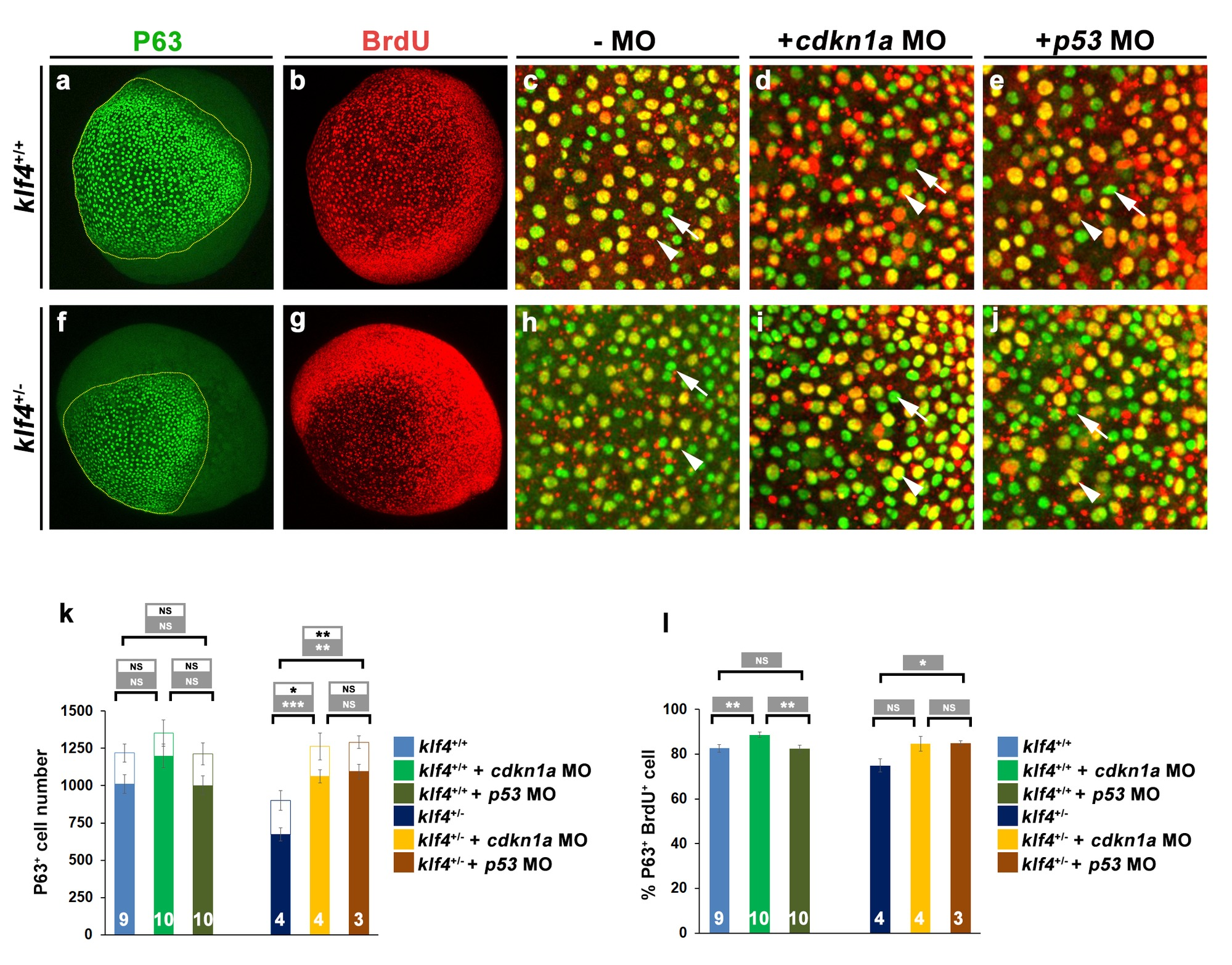

Supplement: S6 Fig — BrdU and p63 colabeling was performed on klf4+/+ or klf4+/- embryos that were uninjected (a-c, f-h), or injected with cdkn1a MO (d, i) or p53 MO (e, j) at bud stage. Examples of p63 and BrdU colocalized (arrowhead) or non colocalzed (arrow) cells are shown. Both p63+ and p63+BrdU+ cell numbers were enumerated in the circled area of embryos under different treatments. Quantification of p63+ cell numbers (open bars) or p63+BrdU+ cell numbers (filled bars) are shown (k). Quantification of the percentage of p63+BrdU+ cells are shown (l). Statistical significance was determined by Student’s t-test. NS, not significant; *p < 0.05; **p < 0.01; ***p < 0.001. Error bars indicate standard error. (TIF) [file pgen.1008058.s006.tif]

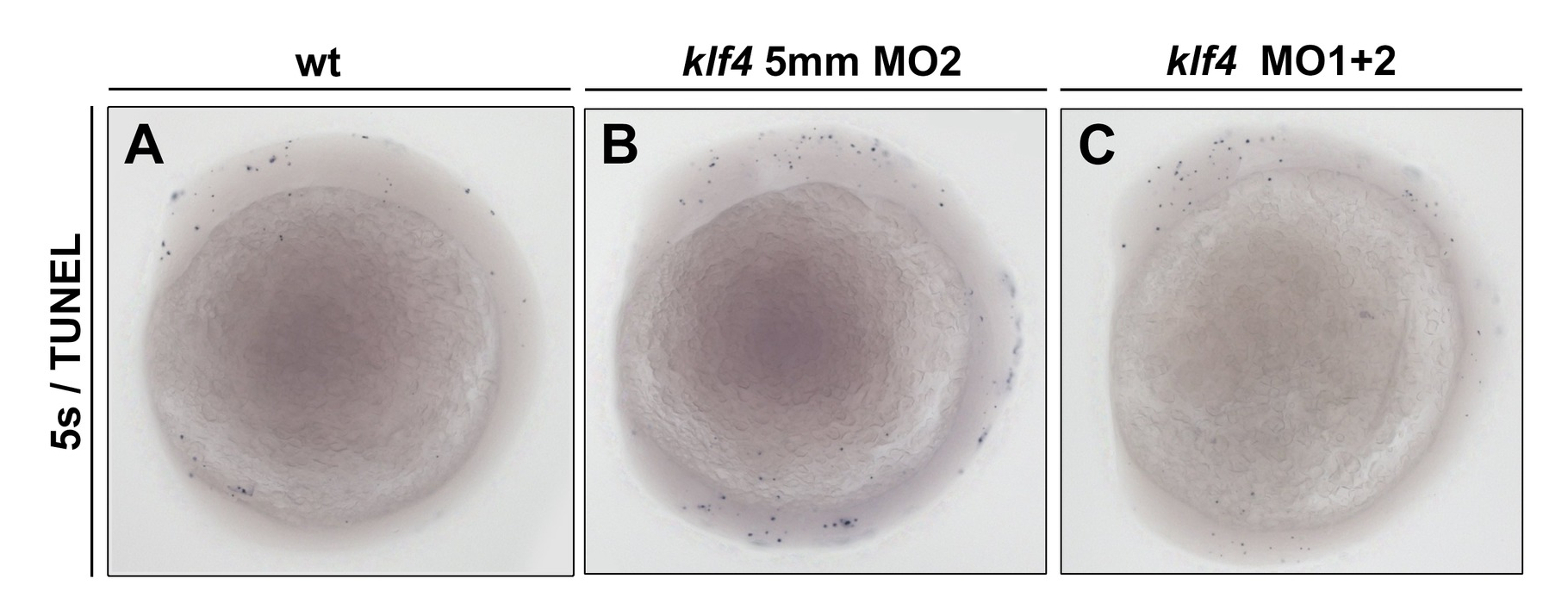

Supplement: S7 Fig — TUNEL staining was not detected in the ventral ectoderm of wild-type (A), embryos injected with either klf4 5mmMO2 (B), or combined klf4 MO1 and klf4 MO2 (C) at 5s stage. (TIF) [file pgen.1008058.s007.tif]

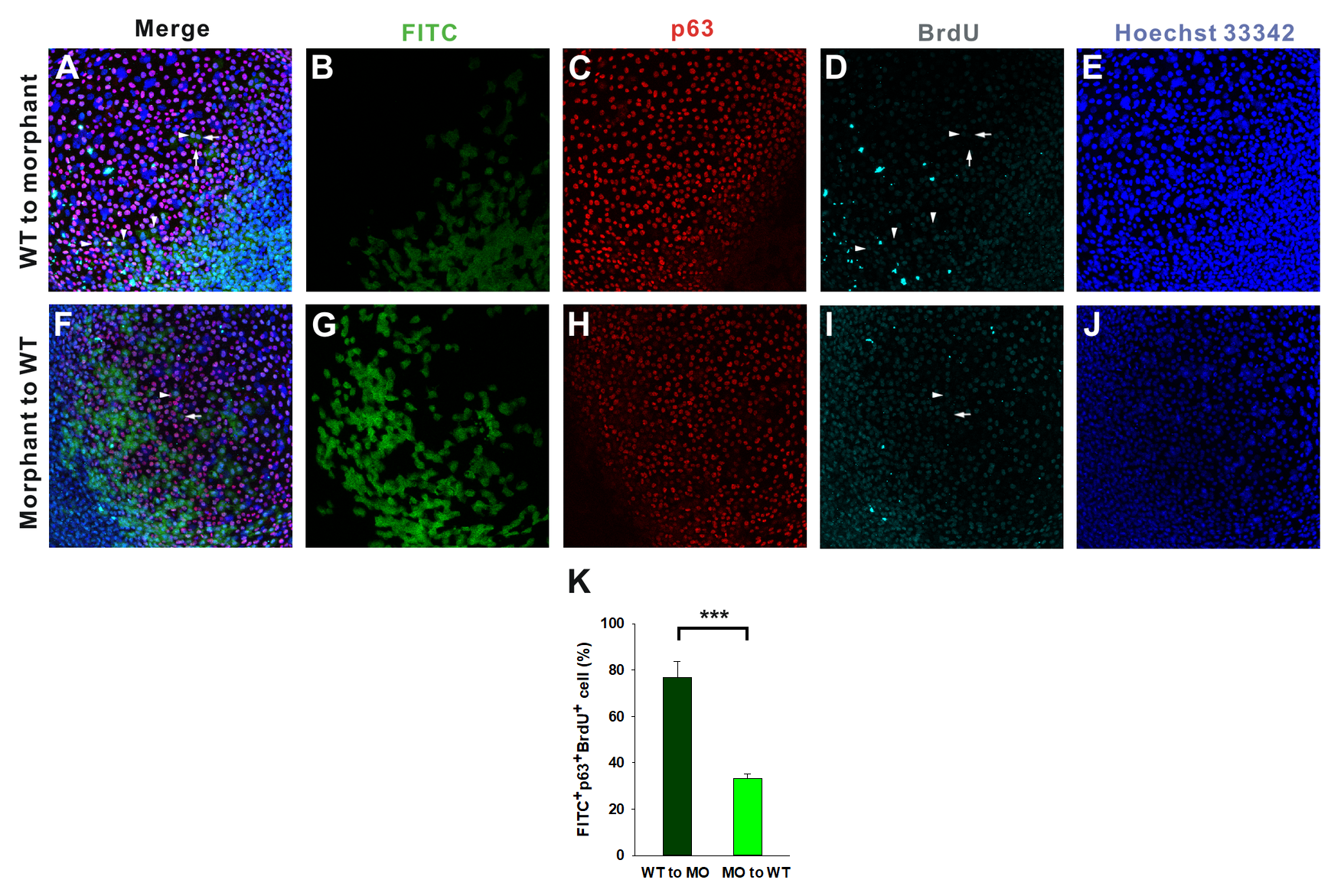

Supplement: S8 Fig — Representative images of chimeric embryos generated by transplantation of fluorescein dextran-labeled wild-type blastomeres into a klf4-morphant host (A-E), or klf4-morphant blastomeres into a wild-type host (F-J). Bud stage embryos were stained with anti-FITC, anti-p63 and anti-BrdU antibodies. Nuclei are counterstained with Hoechst 33342 (E, J). Arrowheads indicate FITC+p63+BrdU+ (A, D, F, I) cells, while arrows indicate FITC+p63+ (A, D, F, I) cells. Quantification of percentage of FITC+p63+BrdU+ cells in these chimeric embryos is shown (K). Student’s t-test. ***p < 0.001. Error bars indicate standard error. (TIF) [file pgen.1008058.s008.tif]

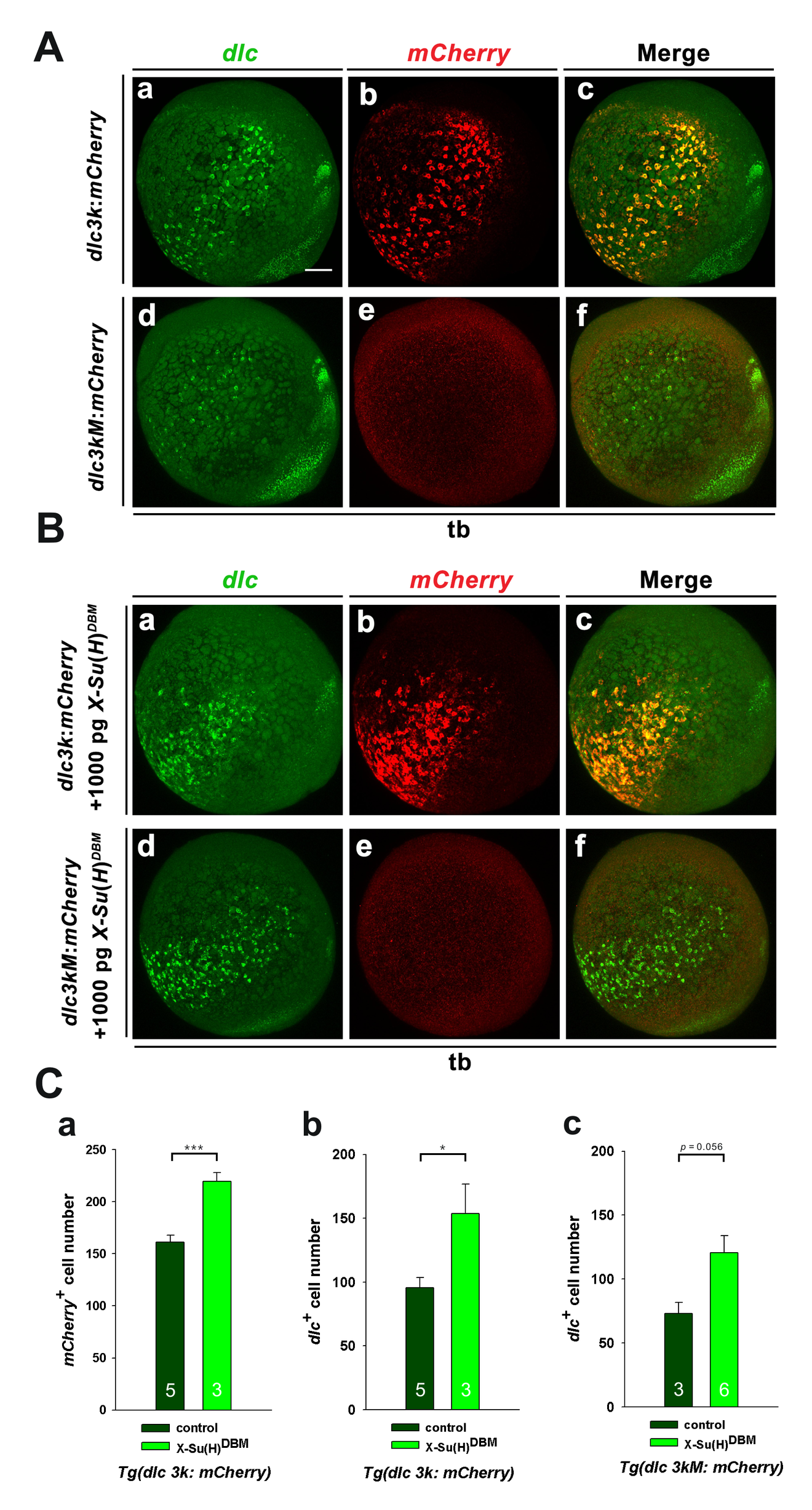

Supplement: S9 Fig — (A) Images of Tg(dlc3k:mCherry) (a-c) and Tg(dlc3kM:mCherry) (d-f) embryos at bud stage. dlc and mCherry signals were detected by in situ hybridization. (B) X-Su(H)DBM-injected Tg(dlc3k:mCherry) (a-c) or Tg(dlc3kM:mCherry) (d-f) embryos are shown. Because the dlc3k and dlc3kM promoter sequences both contain 296 bp 5′ untranslated region of dlc mRNA, each fragment can be hybridized with dlc RNA probe containing full-length cDNA. Similar patterns of mCherry+ and dlc+ ionocyte progenitors were found in both transgenic lines. (C) mCherry+ or dlc+ cell numbers were quantified in Tg(dlc3k:mCherry) or X-Su(H)DBM-injected Tg(dlc3k:mCherry) embryos (a, b). Quantification of dlc+ cell number in Tg(dlc3kM:mCherry) or X-Su(H)DBM-injected Tg(dlc3kM:mCherry) embryos is shown (c). Student’s t-test. *p < 0.05; ***p < 0.001. Scale bar, 100 μm. Error bars indicate standard error. (TIF) [file pgen.1008058.s009.tif]

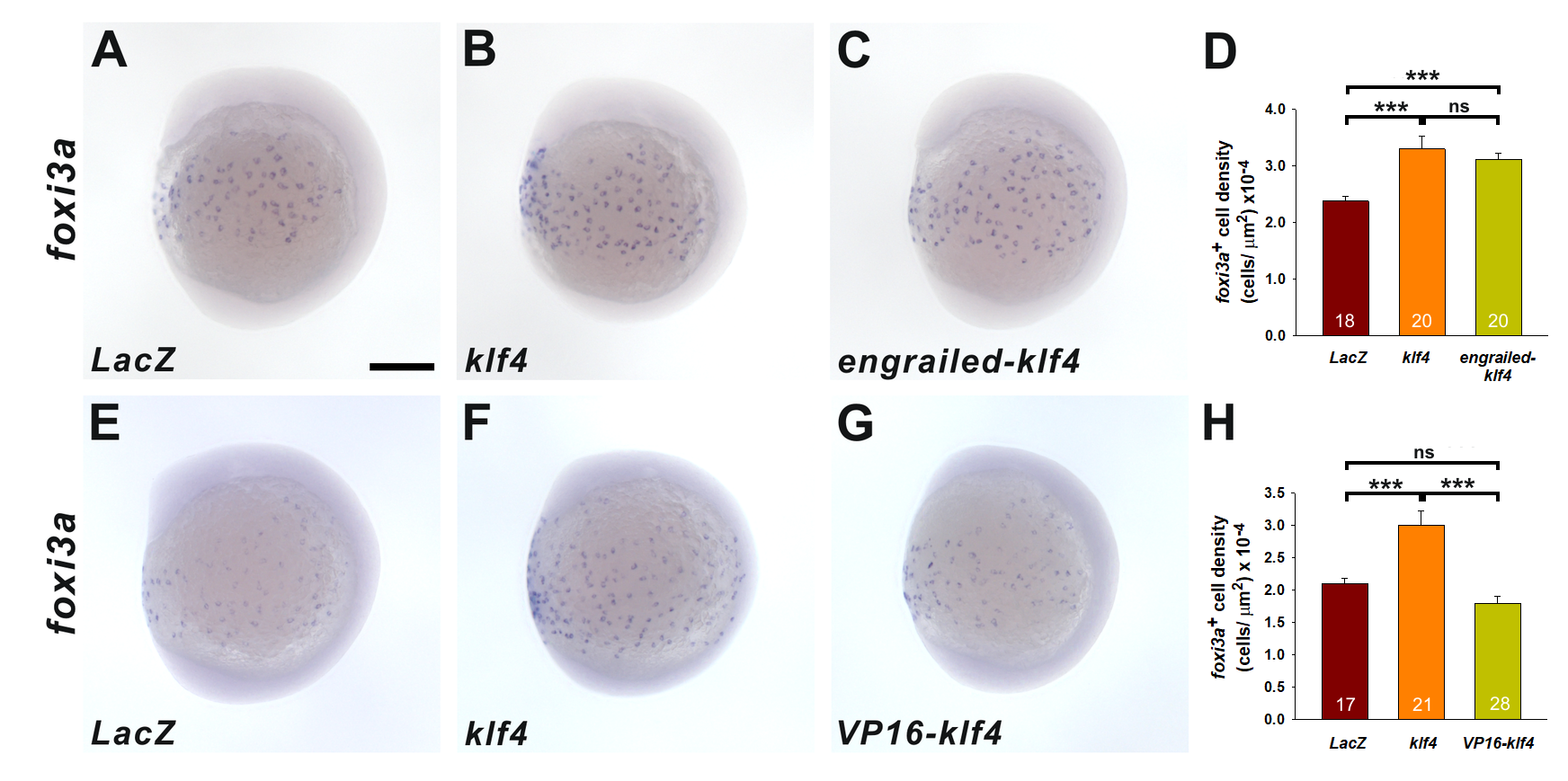

Supplement: S10 Fig — LacZ, klf4 or engrailed-klf4-injected embryos were hybridized with foxi3a antisense RNA probe at 5s stage (A-C). Cell density of foxi3a+ ionocytes in different treatment groups from one representative experiment is shown in the graph (D). Images of LacZ, klf4 or VP16-klf4-injected embryos hybridized with foxi3a antisense RNA probe at 5s stage are shown (E-G). Cell density of foxi3a+ ionocytes in different treatment groups from one representative experiment is shown in the graph (H). Student’s t-test. NS, not significant; ***p < 0.001. Scale bar, 200 μm. Error bars indicate standard error. (TIF) [file pgen.1008058.s010.tif]

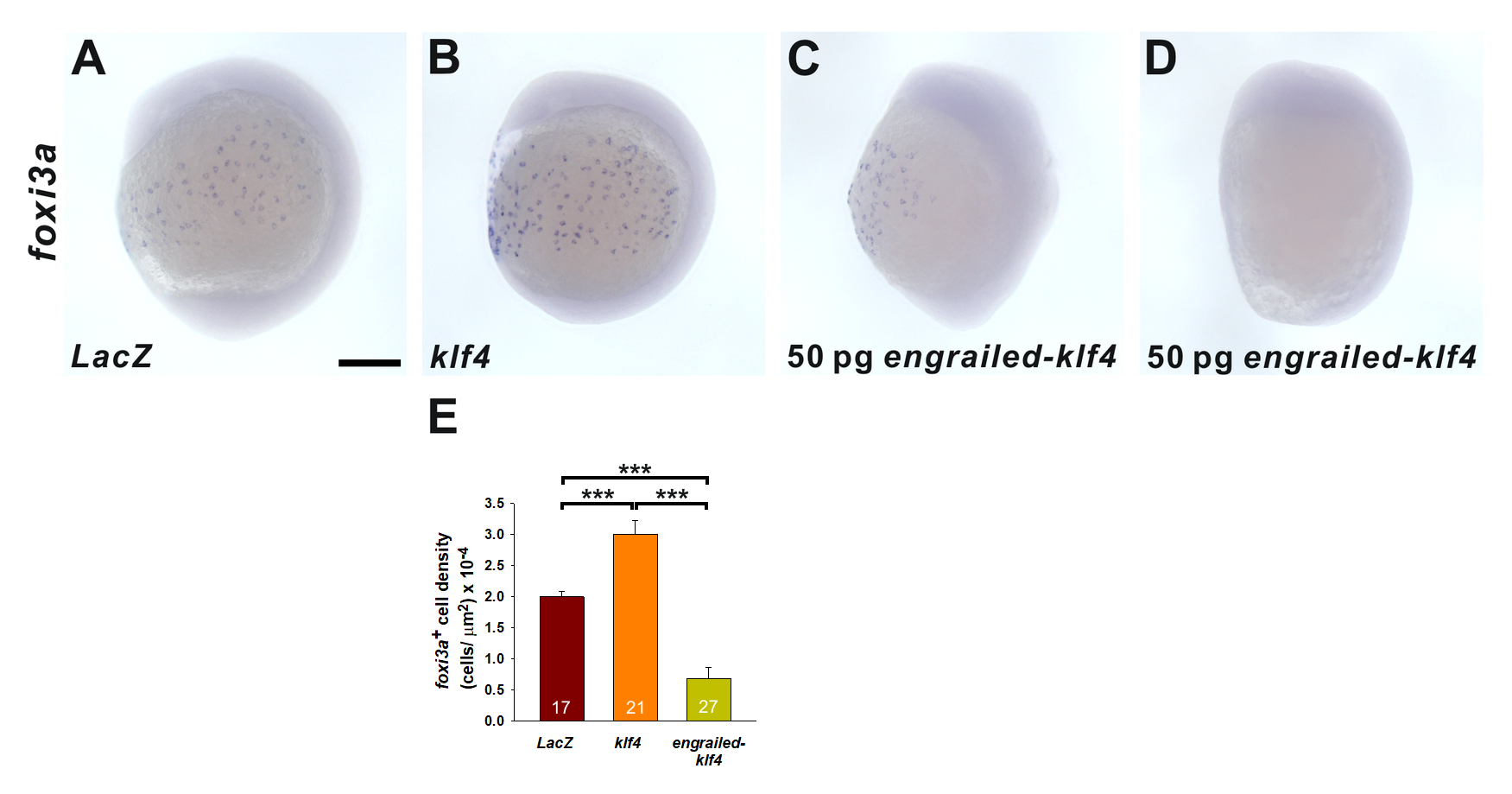

Supplement: S11 Fig — LacZ, klf4 or engrailed-klf4-injected embryos were hybridized with foxi3a antisense RNA probe at 5s stage (A-D). Cell density of foxi3a+ ionocytes in the yolk balls of different treatment groups from one representative experiment is shown in the graph (E). Student’s t-test. ***p < 0.001. Scale bar, 200 μm. Error bars indicate standard error. (TIF) [file pgen.1008058.s011.tif]

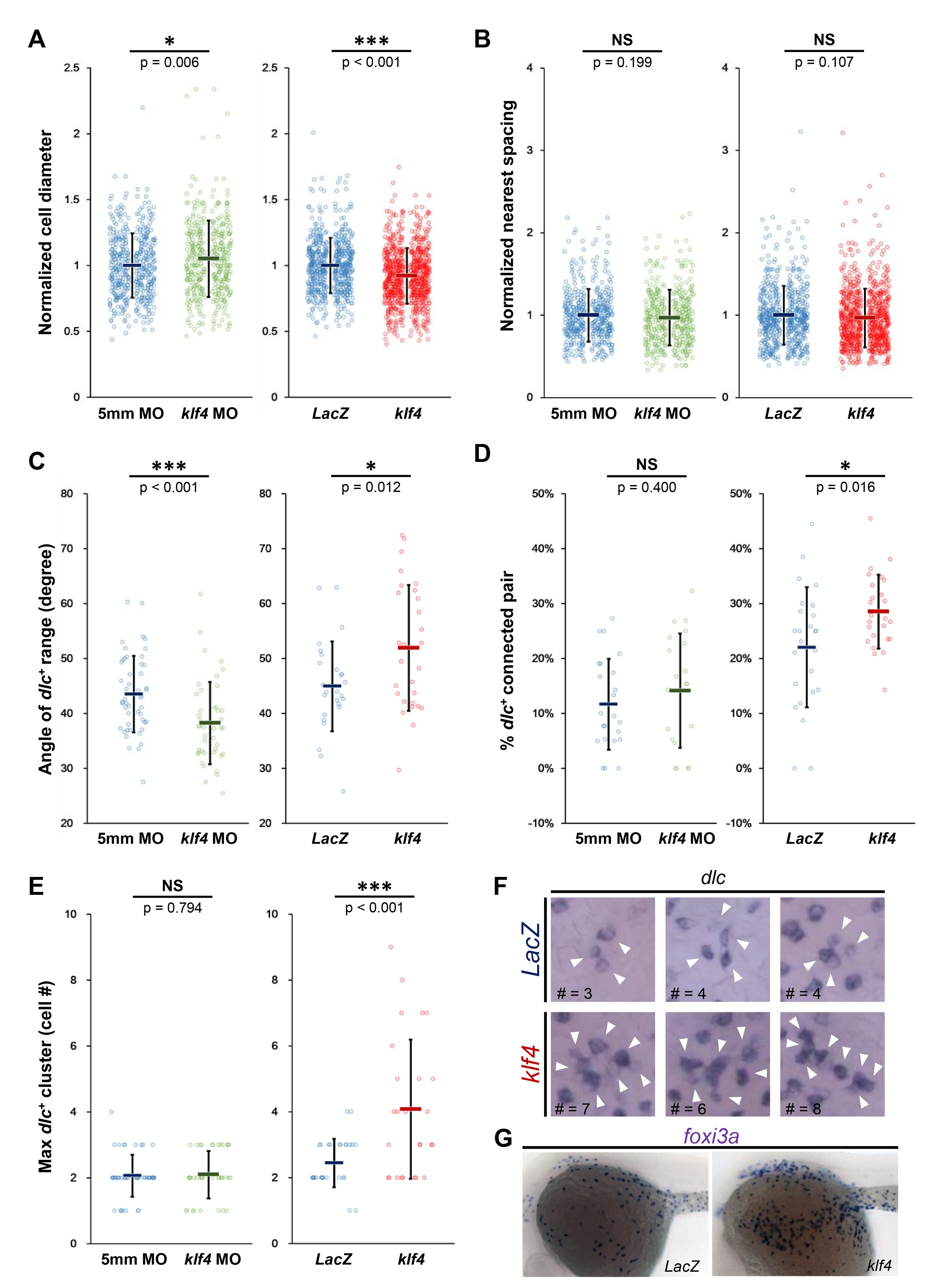

Supplement: S12 Fig — (A) Cell diameter of dlc+ cells was altered by both loss (green) and gain (red) of klf4 function. Values were normalized to control (blue), and individual measurements from embryos (n ≥ 20) are depicted in circles. (B) Nearest spacing of dlc+ cells were not altered by perturbing klf4 expression. The nearest distances between dlc+ cells in the central area (within 40% of embryo diameter) of each embryo were measured in units defined by the average cell diameter, i.e. real distance divided by cell diameter. The normalization and color coding are identical to (A). (C) The range of ionocyte domain was altered, as measured by the angle between two vectors originating at the embryo centroid and extending to the edges of the dlc+ domain. (D) The percentage of connected dlc+ cell pairs was increased by klf4 overexpression. A connected pair is defined as the distance between 2 dlc+ cells being less than 1.25-cell diameters. (E) Maximum dlc+ cells cluster number of embryos is increased by klf4 overexpression. Cell clusters are defined by number of cells that form contiguous pairs. An isolated cell (nearest distance > 1.25-cell diameter long) is cluster number 1, a paired cell has cluster number 2, A cluster of three has cluster number 3, and so on. (F) Representative images from klf4 overexpression in (E). Three images of different embryos from control and klf4 mRNA overexpression groups were selected to show the maximum cluster numbers found. Arrowheads indicate dlc+ cells; # = cluster number. All measurements were made from the same data sets in Fig 3A. (G) Representative images show foxi3a+ cell clusters on the yolk ball in klf4-overexpressing embryos at 24 hpf. Embryo heads to the left. Statistical significance was determined by Student’s t-test. NS, not significant; *p < 0.05; ***p < 0.001. Error bars indicate standard deviation. (TIF) [file pgen.1008058.s012.tif]

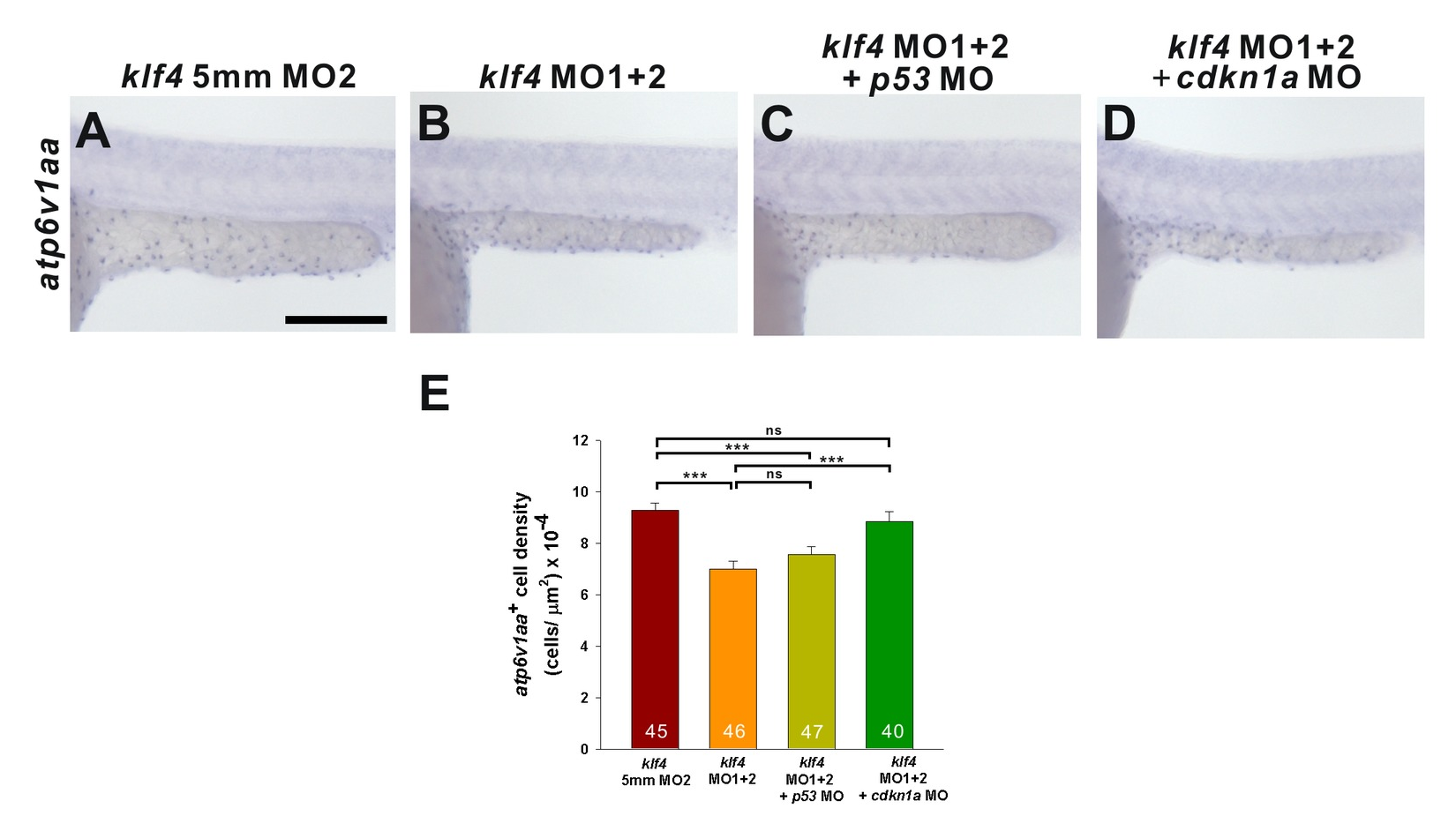

Supplement: S13 Fig — Images are shown of yolk extensions from embryos injected with klf4 5mm MO2 (A), combined klf4 MO1 and klf4 MO2 (B), combined p53 MO/ klf4 MO1/klf4 MO2 (C), or combined cdkn1a MO/ klf4 MO1/klf4 MO2 (D) after hybridization with atp6v1aa antisense RNA probe at 24 hpf. Cell density of atp6v1aa+ ionocytes in the yolk extensions of different treatment groups was quantified (E). Student’s t-test. NS, not significant; ***p < 0.001. Scale bar, 200 μm. Error bar indicates standard error. (TIF) [file pgen.1008058.s013.tif]
